# Identification and Development of Pathogen- and Pest-Specific Defense–Resistance-Associated SSR Marker Candidates Assisted by Machine Learning and Discovery of Putative QTL Hotspots in *Camellia sinensis*

**DOI:** 10.3390/plants15030454

**Published:** 2026-02-02

**Authors:** Ayşenur Eminoğlu

**Affiliations:** Division of Molecular Biology, Department of Biology, Faculty of Art and Science, Recep Tayyip Erdoğan University, 53100 Rize, Türkiye; aysenur.eminoglu@erdogan.edu.tr

**Keywords:** *Camellia sinensis*, simple sequence repeats (SSRs), defense and resistance genes, putative QTL hotspots, marker development, machine learning-assisted prioritization

## Abstract

In this study, a targeted SSR (Simple Sequence Repeat) marker resource was developed based on genes and protein families associated with pathogen- and pest-related defense–resistance mechanisms in *Camellia sinensis*. Forty-one genes and protein families reported to show upregulation, increased expression, or functional validation under disease and pest stress were selected, and the corresponding 195 loci were mapped onto the *Camellia sinensis* cv. Shuchazao genome. SSR screening within gene bodies and gene-flanking regions (±5 kb) identified 5197 SSR loci. Putative QTL hotspot regions were defined using locus-based sliding-window analysis, *Z*-score calculations, and permutation tests, yielding 633 SSRs filtered at the 99% and 95% significance thresholds. Proteome-wide scans based on conserved amino acid motifs identified multiple loci within the WRKY, NAC, LRR, PRX, and CHI families, and Random Forest analysis was used to prioritize SSRs within these families. Finally, 386 SSR primer sets were designed and evaluated by *in silico* PCR across six tea genomes. Of these, 245 primers produced amplicons in more than one genome, and 124 exhibited polymorphic information content values greater than 0.500. Overall, the developed SSR panels represent a biologically contextualized and experimentally transferable marker resource targeting defense–resistance-associated genic and gene-proximal regions.

## 1. Introduction

*Camellia sinensis*, a member of the family *Theaceae*, is a woody, evergreen (non-deciduous) plant with an approximately 4000 Mb genome. The tea plant has two well-known major varieties, var. *sinensis* and var. *assamica*, which are morphologically distinct from each other [1,2]. *Camellia sinensis* represents a strategic agricultural crop cultivated across a wide spectrum of agro-ecological environments and consumed daily by millions of people worldwide [2]. However, the long-term cultivation of tea in dense and genetically homogeneous plantations creates stable microclimatic conditions that favor pests, and rising temperatures together with altered precipitation regimes further intensify pest pressure by increasing the number of generations, feeding rates, and dispersal potential of herbivores and pathogens [3,4,5,6,7,8]. The tea plant accumulates a broad array of defensive metabolites, including catechins, caffeine, flavonoids, and volatile compounds, and activates jasmonic acid and salicylic acid signaling pathways, lignin deposition, and modifications in cuticular wax biosynthesis following biotic attack [9,10,11,12,13,14,15]. Pathogens (e.g., *Colletotrichum* spp.) and specialized herbivores (such as leafhoppers, mites, and aphids) have co-evolved to circumvent or tolerate the plant’s defense machinery, thereby maintaining their high pest status [3,6,9,12,16]. Under such persistent biotic stress, the extensive use of chemical pesticides and fungicides has accelerated the development of resistance in target populations, undermining long-term control efficacy. Furthermore, phytotoxic effects, residue accumulation, and regulatory restrictions have imposed significant constraints on both tea production and trade [4,13,17,18,19,20]. Excessive chemical inputs also exert deleterious effects on soil and aquatic ecosystems, as well as human health [21,22,23].

In this context, genetic resistance constitutes an environmentally sustainable and cost-effective alternative that forms the cornerstone of integrated pest management in tea cultivation [24,25,26]. Field evaluations have documented substantial genotypic variation in resistance to anthracnose, gray leaf blight, blister blight, mites, aphids, and leafhoppers, with resistant cultivars typically exhibiting smaller lesion sizes or reduced insect-induced damage [3,11,27,28,29,30,31].

Moreover, studies on *Camellia sinensis*-specific pathogens have demonstrated that transcription factors belonging to the ethylene response factor (ERF) family and genes involved in jasmonic acid (JA) biosynthesis play positive regulatory roles in anthracnose resistance [9,31]. During infection by the fungal pathogen *Exobasidium vexans*, members of the pathogenesis-related protein 1 (PR-1) gene family, together with salicylic acid (SA) and jasmonic acid (JA)-mediated defense pathways, were found to be strongly activated, and resistance (R) genes exhibited higher expression levels in resistant genotypes [32,33]. Additionally, transcription factors such as WRKY and NAC, along with defense-associated protein families, have been reported to be preferentially expressed in tolerant genotypes [34]. In defense responses against phloem-feeding and chewing herbivores, the upregulation of genes involved in terpenoid biosynthesis, cell wall reinforcement, and the phenylpropanoid pathway has been identified as a key protective mechanism [2,29,35,36]. These studies indicate that resistance responses in tea against different diseases and pests are shaped around common genetic components such as transcription factors, hormone signaling, secondary metabolite biosynthesis, and cell wall modification.

This functional background necessitates the monitoring of genetic variations associated with disease and pest resistance in tea using appropriate molecular markers. Simple sequence repeats (SSRs, microsatellites) are molecular markers composed of short, tandemly repeated DNA motifs; due to their high polymorphism, co-dominant inheritance, multi-allelic nature, genome-wide distribution, and PCR-based detection, they enable the construction of linkage maps for loci associated with disease resistance, quantitative trait locus (QTL) mapping, and the implementation of routine marker-assisted selection (MAS) even in resource-limited laboratories [37,38,39,40,41,42,43,44,45].

Traditional SSR marker development approaches rely on subjective primer or marker screening and are time-consuming, costly, and inconsistent. Although large NGS datasets allow in silico SSR mining, they also yield a large number of non-informative or monomorphic loci that require experimental validation. Therefore, machine learning-based approaches have emerged as auxiliary bioinformatic tools that evaluate primer and motif features simultaneously to facilitate the selection or prioritization of more informative SSR loci for specific applications [46].

Despite the availability of genome-wide SSR markers for tea, most resources are designed for general genotyping or diversity studies rather than being explicitly structured around pathogen- and pest-associated defense loci; therefore, they do not provide systematically organized, prioritized SSR primer panels that can be directly applied to resistance screening targeting specific pathogen- or pest-associated regions. To address this gap, we developed the first comprehensive and integrative SSR primer resource centered on defense- and resistance-associated targets, based on genes previously demonstrated in the literature to be involved in defense and resistance through transcriptomic and gene expression evidence as well as functional and biological characterization, and integrating locus-centered pQTL hotspot analysis, permutation-based enrichment testing, and machine learning-assisted prioritization.

## 2. Results

### 2.1. Targeted Defense-Resistance-Associated Genes and Loci

In this study, SSRs were identified by targeting a total of 41 distinct gene and protein families (Table 1) that have been reported in the literature to exhibit upregulation or high expression in response to diseases caused by *Colletotrichum fructicola* [9], *Acaphylla theae* Watt (pink tea mite) [3], *Colletotrichum camelliae* [31], *Exobasidium vexans* Massee (blister blight) [32,33,34], *Empoasca vitis* Göthe [29], *Empoasca onukii* Matsuda [35,36], and *Ectropis obliqua* [36], together with their corresponding 195 unique loci (Supplementary File S1: Final SSR catalogue). The names of the gene or protein families listed in Table 1 were obtained from annotations of the AHAU_CSS_1 genome, with the exception of *CsERF105* and *CsOPR3*. For these two genes, nomenclature was assigned based on functionally characterized gene names reported in the literature, rather than on the reference genome annotations used in this study.

In addition, conserved amino acid motifs belonging to WRKY, NAC, LRR, peroxidase (PRX), and chitinase (CHI) families, which have been reported to be upregulated under *Exobasidium vexans* infection conditions [34], were systematically screened across the entire AHAU_CSS_1 genome for the first time. As a result of this amino acid motif-based proteome-wide analysis, members of the corresponding protein families harboring the same amino acid motifs were identified, and SSRs were developed from these corresponding loci (Supplementary File S2: Final target amino acid motif-based loci). Furthermore, the proteome-wide positional distributions of these amino acid motifs were mapped to generate amino acid motif atlases, which are presented in Supplementary File S3 (proteome-wide amino acid motif atlas).

### 2.2. Genomes and Assemblies

The genome assemblies used for SSR mining, primer design, and in silico PCR analyses are presented in Table 2.

### 2.3. SSR-Identified Loci

As a result of the initial screenings, SSRs were identified at a total of 5197 loci in the first stage (Supplementary File S4. Initial SSR screening output).

### 2.4. Final SSR Panel and pQTL Hotspots

**Figure 1:**
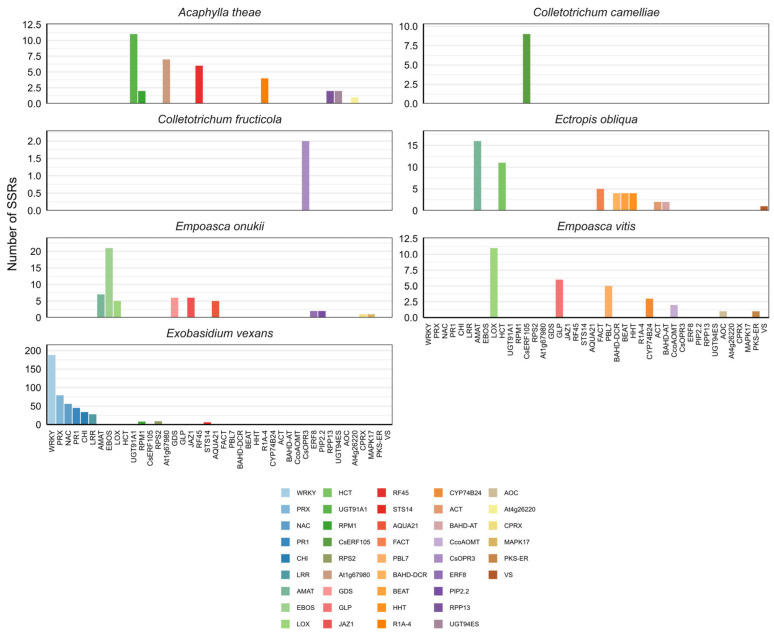
Distribution of SSRs across defense–resistance-related genes or protein families responding to different pathogens and pests. Each panel shows the number of SSRs identified for resistance-associated genes and gene families corresponding to the relevant pathogen or pest species. SSR abundance varies strongly across pathogens and pests, with *Exobasidium vexans* showing the highest SSR counts.

Following *Z*-score filtering, permutation analysis was performed, and SSRs with the highest structural suitability were selected for each locus, leading to the construction of a final SSR panel based on the 99% permutation threshold. Accordingly, as a result of the selection process conducted separately for seven pathogens and pests and 41 gene and protein family targets, a total of 633 SSRs were identified (Figure 1 and Supplementary File S1: Final SSR catalog).

When the distribution of the 633 identified SSRs was examined by motif type, the highest frequency was observed for dinucleotide repeats (*n* = 366). This was followed by hexanucleotide (*n* = 104) and pentanucleotide (*n* = 87) repeats, respectively. Trinucleotide (*n* = 50) and tetranucleotide (*n* = 26) motifs were represented at lower frequencies (Supplementary File S5: Summary statistics for motif classes, repeat distributions, and distance metrics).

When *Z*-scores calculated according to motif length were examined, tetranucleotide motifs exhibited the highest mean and median *Z*-score values (*M* = 2.965; *Md* = 3.018), indicating that this motif group displays statistically the highest mean/median values among motif classes. Although dinucleotide motifs constituted the numerically dominant group (*n* = 366), they demonstrated *Z*-score performance comparable to that of tetranucleotide motifs (*M* = 2.932). Tri-, penta-, and hexanucleotide motifs showed closely similar mean and median *Z*-score values (Supplementary File S5: Summary statistics for motif classes, repeat distributions, and distance metrics).

When the distribution of SSRs was examined according to pathogen and pest species, they were found to be clearly distributed in accordance with the locus density targeted in the study. The highest number of SSRs was detected in loci associated with *Exobasidium vexans* (*n* = 453). Notable numbers of SSRs were also identified for the leafhopper *Empoasca onukii* (*n* = 56), the lepidopteran pest *Ectropis obliqua* (*n* = 49), and the pink tea mite *Acaphylla theae* (*n* = 35). In contrast, *Empoasca vitis* (*n* = 29) showed a moderate level of representation, whereas only nine SSRs were identified for the anthracnose pathogen *Colletotrichum camelliae* (*n* = 9) (Supplementary File S5: SSRs per pathogen/pest).

**Figure 2:**
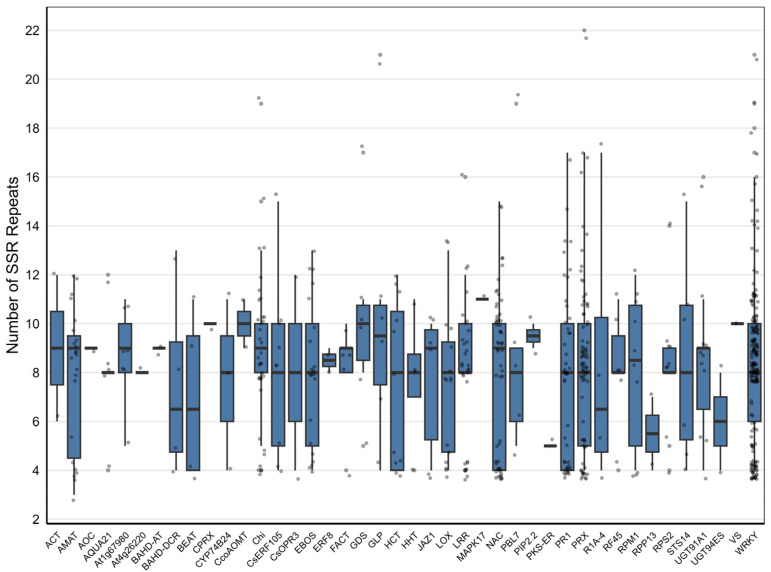
Distribution of SSR repeat numbers across defense- and resistance-associated genes and protein families. Boxes represent the median, interquartile range, and extreme values of SSR repeat numbers for each gene or protein family. Grey points indicate individual SSRs.

When SSR repeat characteristics were evaluated across defense–resistance-related genes and protein families, WRKY, PRX, NAC, CHI, and LRR exhibited the highest numbers of SSRs and similar mean repeat values. In these groups, mean repeat numbers were generally within the 8–9 range. SSR motifs derived from other targeted genes represented by a single locus showed mean repeat numbers ranging approximately between 7 and 11 (Figure 2).

As shown in Figure 2, dinucleotide motifs predominate across nearly all genes. Protein families identified through proteome-wide screening based on conserved amino acid motifs (WRKY, PRX, NAC, LRR, and CHI) exhibit closely similar median repeat values and homogeneous variation profiles. In contrast, genes or proteins represented by a single or limited number of loci show narrower distributions and more stable repeat numbers. Overall, the SSR repeat profiles across genes and protein families show distinct distribution patterns across multi-locus families and single-locus targets (Supplementary File S5: Repeats per gene).

Correlation analyses indicate that there is no significant linear or rank-based association between SSR repeat number and *Z*-scores (Pearson *r* = −0.024; Spearman *ρ* = −0.018; *n* = 633) (Supplementary File S5: Correlation analyses).

**Figure 3:**
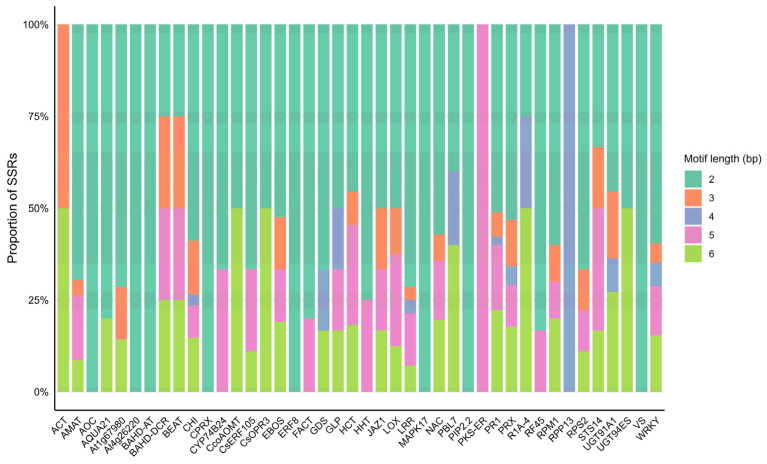
Distribution of SSR motif lengths across defense- and resistance-associated genes and protein families. Stacked bar charts show the relative proportions of SSRs identified for each gene or protein family according to motif length (2–6 bp). Motif-length composition differs among targets, indicating gene or protein family-specific enrichment patterns in SSR motif-length classes.

Analysis of SSR motif lengths across defense- and resistance-associated genes and protein families revealed a clear predominance of short motifs, particularly di- and trinucleotide repeats, throughout the entire dataset. However, multi-locus protein families identified through proteome-wide amino acid motif–based screening (WRKY, PRX, NAC, LRR, and CHI) exhibited a more heterogeneous distribution of SSR motif lengths. In contrast, genes represented by a single locus or a limited number of loci showed a pronounced predominance of specific motif lengths (Figure 3 and Supplementary File S5: SSR motif length per gene and protein family).

**Figure 4:**
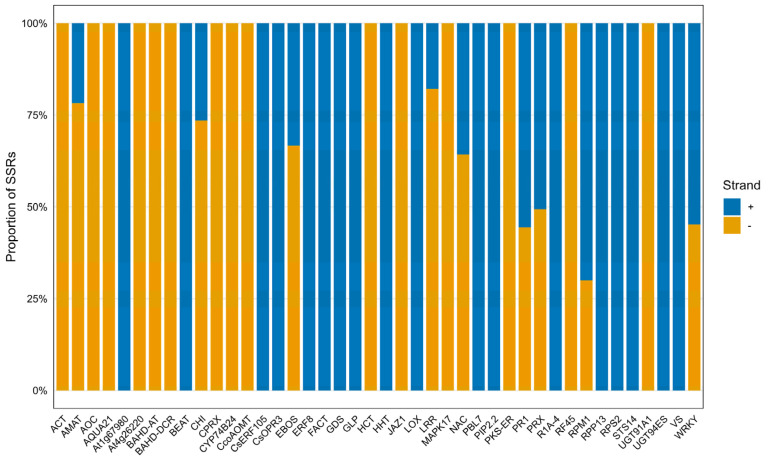
Proportional distribution of SSRs identified across the + and − DNA strands in defense- and resistance-associated genes and gene–protein families. Each bar represents the relative proportions of SSRs located on the positive (+) and negative (−) strands for the corresponding gene or protein family.

When the strand orientation of SSRs was examined, no pronounced orientation bias between the + and − strands was observed in the majority of genes and gene families (Figure 4 and Supplementary File S6: Strand orientation).

When the locus–SSR distances calculated for the 633 identified SSRs were examined, the mean distance was 1437 bp, while the median distance was 874 bp. A minimum distance of 0 bp indicates that a substantial proportion of SSRs are located directly within genic regions. In contrast, the maximum distance of 4970 bp, together with a high standard deviation (*SD* = 1512 bp), demonstrates that SSRs are widely distributed across both intragenic regions and gene-proximal intergenic regions. This distribution indicates that the SSRs identified in this study are not confined to coding regions but also encompass gene-flanking regions with potential regulatory relevance (Supplementary File S6: Distance stats).

In addition to SSRs located within genic regions, SSRs were also identified in gene-proximal regions within the predefined pQTL windows. When the average SSR density per QTL window is considered, short-motif SSRs, particularly dinucleotide repeats, are enriched at high repeat numbers (≥8 repeats), whereas longer-motif SSRs and lower-repeat variants show low densities within resistance. When the genomic distribution of the 633 identified SSRs was evaluated at both major region and sub-region levels, the markers were found to be predominantly concentrated in gene-proximal and functionally critical regions. A total of 196 SSRs were located within genic regions, all of which corresponded to intronic SSRs (*n* = 196). Of the 437 SSRs positioned in intergenic regions, the majority were clustered within gene-proximal regulatory areas, showing particularly high densities in promoter upstream (*n* = 203) and downstream (*n* = 229) regions. In contrast, the very limited number of SSRs located in distal intergenic regions (*n* = 5) clearly indicates that the SSRs were not selected from random genomic locations, but rather from functionally relevant regions associated with genes within pQTL intervals (Supplementary File S6: Genic and intergenic SSR distribution).

**Figure 5:**
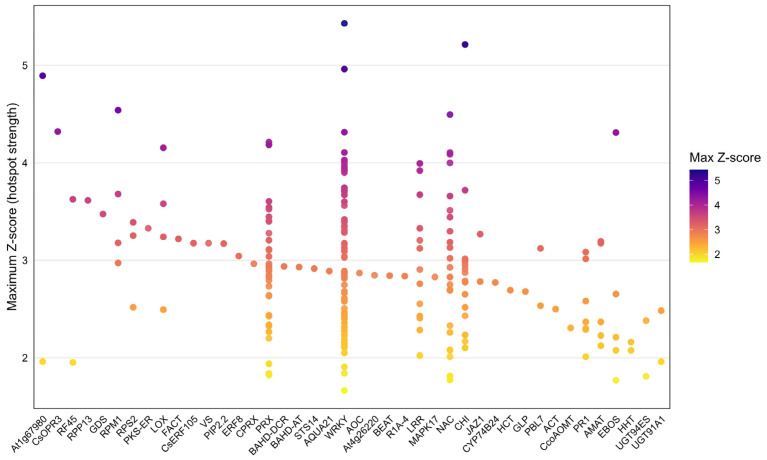
Distribution of maximum *Z*-scores (hotspot signal strength) of SSRs associated with defense–resistance-related genes and protein families. Each point represents the maximum *Z*-score of an individual SSR locus identified within the corresponding gene or protein family. Point color reflects the maximum *Z*-score value, where higher *Z*-scores indicate stronger hotspot signals.

In Figure 5, *Z*-scores reflect the maximum signal intensity calculated across pQTL windows, with higher values indicating stronger hotspot contributions. Protein families identified through amino acid motif-based proteome-wide screening and represented by multiple loci (e.g., WRKY, PRX, NAC, LRR, and CHI) exhibit a broad *Z*-score distribution and high variance, whereas genes represented by a limited number of loci show *Z*-scores clustered within a narrower range.

When the physical positions of SSRs and genes across pQTL regions were mapped while preserving true genomic distances, SSRs and their corresponding genes were co-localized within the same pQTL intervals. To illustrate this spatial organization, pQTL boundaries, locus locations, and SSR positions along scaffolds are presented on a normalized scale (Norm Max = 1000) while preserving true physical distances. This representation visualizes the distribution of SSRs across genic and locus-proximal regions within pQTL intervals, as well as their spatial organization along the same scaffold (Supplementary File S5: SSR per pQTL; Supplementary File S6: SSR per pQTL coordinates). Detailed physical maps for all scaffolds are provided in Supplementary File S7.

### 2.5. Random Forest Ablation Analysis

Using the same selection rule (selecting the top-5 SSR candidates per locus) within the WRKY family, it was observed that the ML-based prioritization (RF, prob_pos ranking) provided a markedly higher enrichment of hotspot-positive SSRs compared to the baseline heuristic ranking (repeat number + distance to gene) (positive rate = 0.964; 188/195; baseline: positive rate = 0.488; 179/367), corresponding to an uplift of +0.476 (Supplementary File S8: Random Forest ablation analysis results).

### 2.6. General Characteristics of the Developed SSR Primer Sets

As a result, 386 SSR primer sets compatible with standard PCR conditions and directly transferable to experimental applications were obtained for the developed SSRs (Supplementary File S9: Final SSR primer dataset). The highest numbers of loci (*n* = 154) and SSR primers (*n* = 278) were detected in genes associated with *Exobasidium vexans*, a major fungal pathogen of tea (Table 3).

Among pathogen and pest species, a total of 33 SSR primers were developed from 12 loci for *Empoasca onukii*, while 31 SSR primers were developed from 13 loci for *Ectropis obliqua*. The number of primers developed for other pest species is provided in Table 3. Of the obtained SSR primers, 244 produced two or more amplicons at the target loci, whereas the remaining 142 primers yielded a single amplicon (Supplementary File S10: SSR primer locus per pathogen and pest).

**Figure 6:**
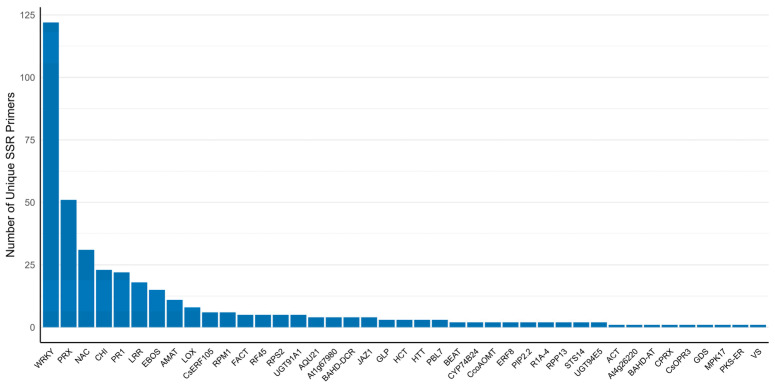
Number of unique SSR markers (primers) designed for each gene and protein family located within pQTL regions. Bars are ordered from highest to lowest, indicating uneven primer representation across targets.

When they were evaluated across gene and protein family targets, it was observed that the number of unique SSR primers was distributed in proportion to the number of loci targeted in the study (Figure 6 and Supplementary File S10: Summary of unique SSR primers by gene and protein family).

### 2.7. In Silico PCR Results

The *in silico* PCR outputs generated across a total of six genomes were organized and exported to include primer-specific individual sheets, as well as summary and aggregated result tables (Supplementary File S11: Multi-genome *in silico* PCR outputs). The *in silico* PCR analyses revealed that the number of primers producing amplicons in different tea cultivars varied in a cultivar-dependent manner. Accordingly, apart from the Shuchazao cultivar for which the primers were originally designed, the number of primers yielding products in other cultivars was as follows: Tieguanyin (*n* = 178), UPASI-3 (*n* = 168), TES-34 (*n* = 163), L618 (*n* = 156), and TV1 (*n* = 123).

When the polymorphic information content (PIC) of the markers was examined, 124 primers were found to have PIC values > 0.500. In contrast, 142 primers exhibited a PIC value of 0.00 and produced amplicons only in the AHAU_CSS_1 genome (Supplementary File S12: SSR primers’ PIC analyses). Accordingly, SSR primers with PIC values > 0.500 are provided in Supplementary File S13 (High PIC SSR primers (PIC > 0.500)).

**Figure 7:**
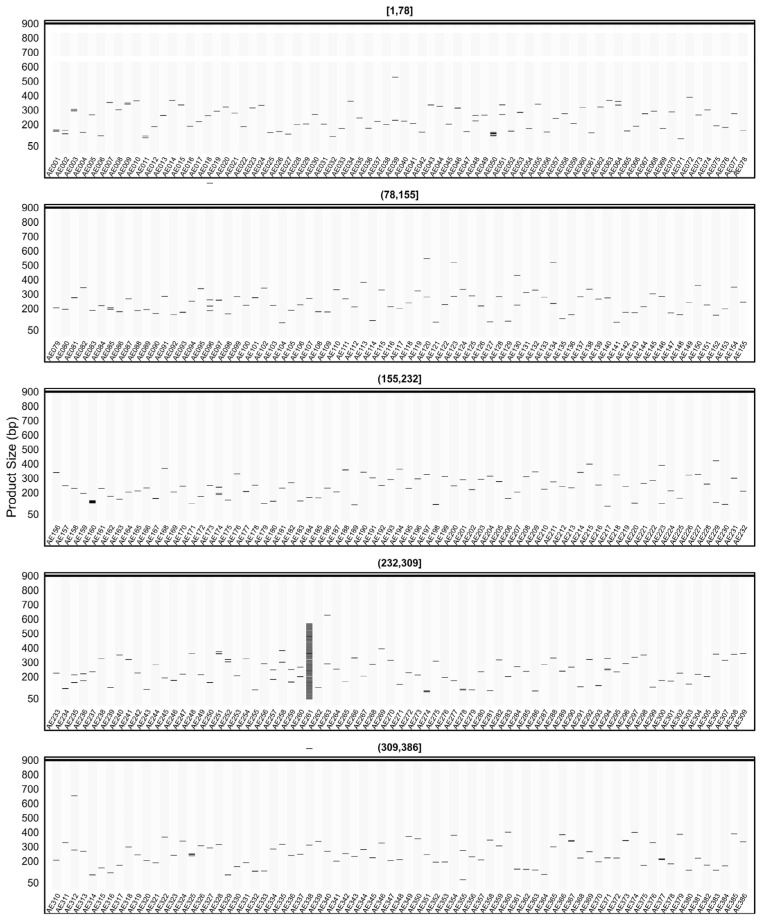
Virtual AGE figure of the *in silico* PCR results of defense- and resistance-associated SSR primers generated on the AHAU_CSS_1 genome. The numerical ranges shown above each panel represent primer indices. Each horizontal band represents an in silico amplicon predicted for the corresponding SSR primer, and the y-axis indicates the expected product size (bp). Multiple bands indicate multi-locus amplification.

Figure 7 presents the amplicon patterns obtained from the *in silico* PCR analysis of SSR primer sets designed from pQTL regions on the AHAU_CSS_1 genome. The results, displayed in five panels, reflect the expected product sizes and specificity profiles of different primer groups. One of the example primer AE261, designed from the SSR region selected for *CcoAOMT*, was observed to generate 88 distinct amplicons in the AHAU_CSS_1 genome outside the targeted loci. In addition, amplicons larger than 900 bp are reported in Supplementary File S11 (Multi-genome *in silico* PCR outputs).

**Figure 8:**
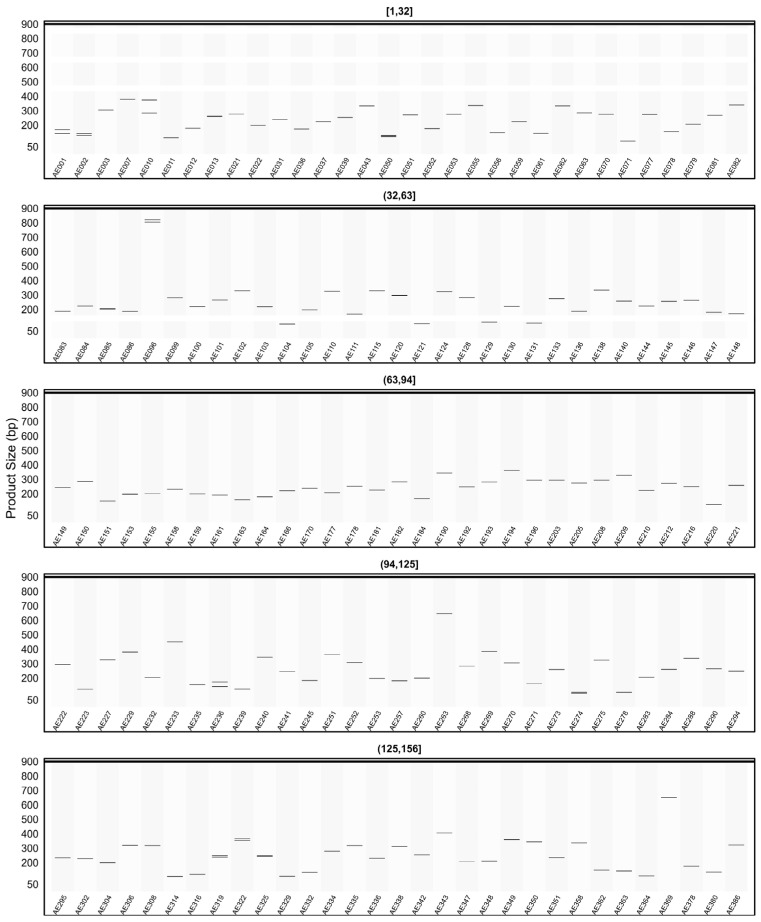
Virtual AGE figure of the *in silico* PCR results of defense- and resistance-associated SSR primers generated on the *Camellia sinensis* L618 genome. The numerical ranges shown above each panel represent primer indices. Each horizontal band represents an in silico amplicon predicted for the corresponding SSR primer, and the y-axis indicates the expected product size (bp). Multiple bands indicate multi-locus amplification.

**Figure 9:**
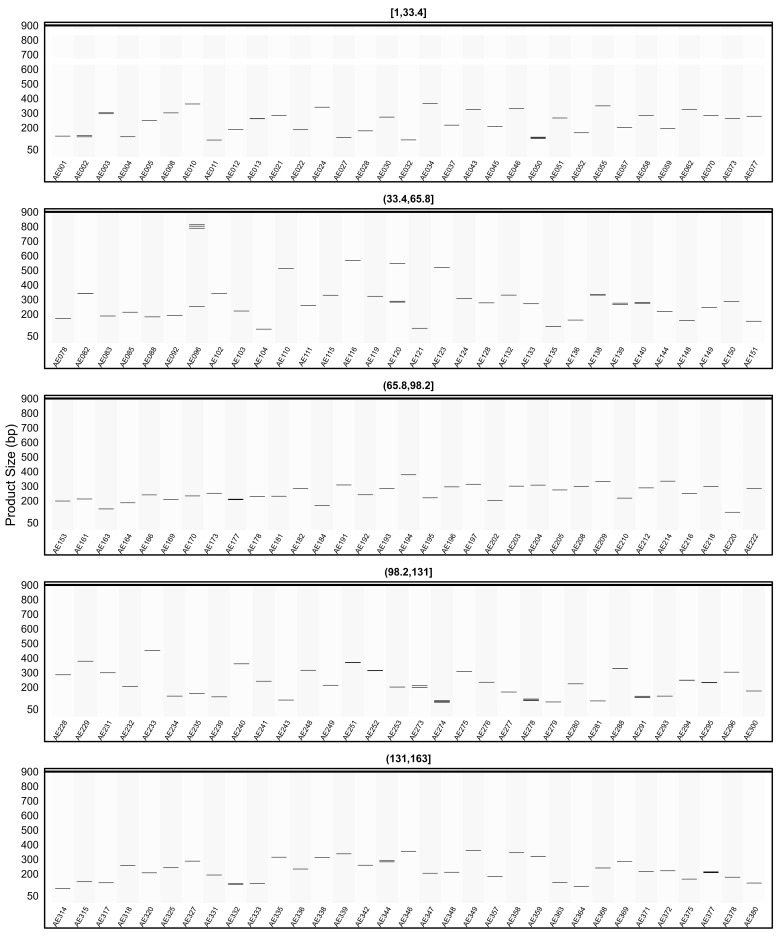
Virtual AGE figure of the *in silico* PCR results of defense- and resistance-associated SSR primers generated on the ASM2053679v1 genome. The numerical ranges shown above each panel represent primer indices. Each horizontal band represents an in silico amplicon predicted for the corresponding SSR primer, and the y-axis indicates the expected product size (bp). Multiple bands indicate multi-locus amplification.

**Figure 10:**
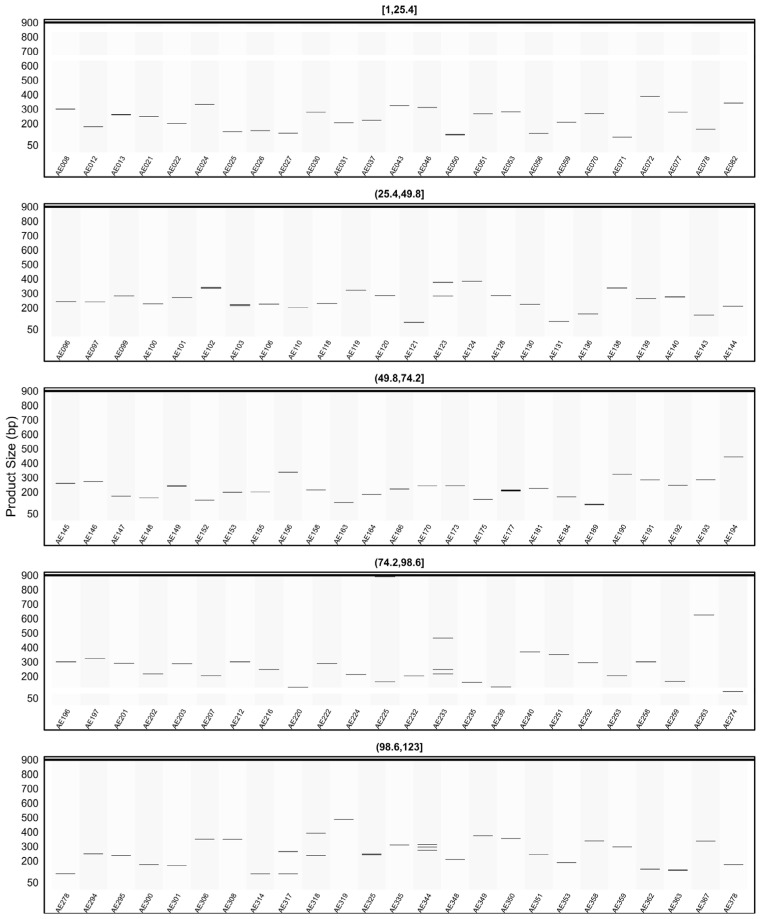
Virtual AGE figure of the *in silico* PCR results of defense- and resistance-associated SSR primers generated on the IND_Tea_TV1 genome. The numerical ranges shown above each panel represent primer indices. Each horizontal band represents an in silico amplicon predicted for the corresponding SSR primer, and the y-axis indicates the expected product size (bp). Multiple bands indicate multi-locus amplification.

**Figure 11:**
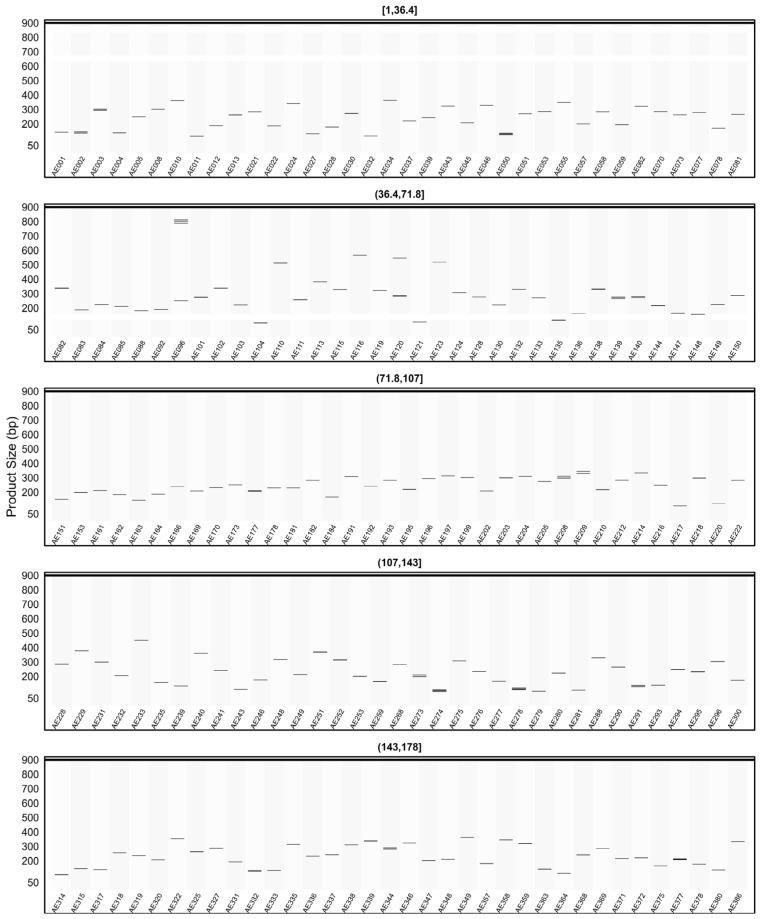
Virtual AGE figure of the *in silico* PCR results of defense- and resistance-associated SSR primers generated on the ASM1731120v1 genome. The numerical ranges shown above each panel represent primer indices. Each horizontal band represents an in silico amplicon predicted for the corresponding SSR primer, and the y-axis indicates the expected product size (bp). Multiple bands indicate multi-locus amplification.

**Figure 12:**
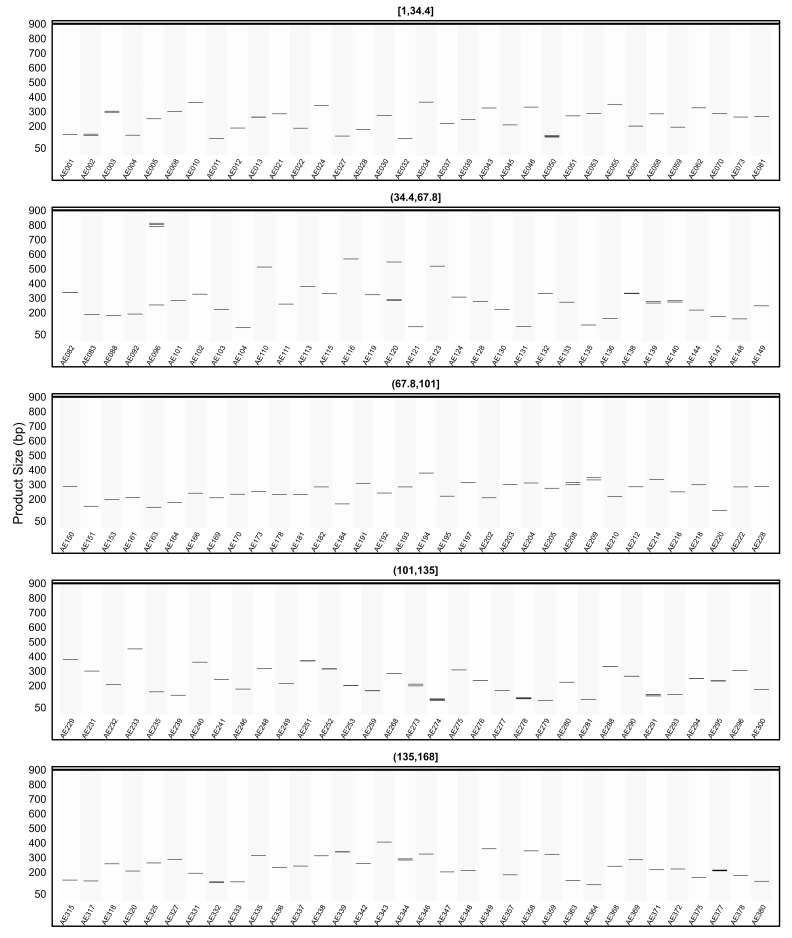
Virtual AGE figure of the *in silico* PCR results of defense- and resistance-associated SSR primers generated on the ASM2053686v1 genome. The numerical ranges shown above each panel represent primer indices. Each horizontal band represents an in silico amplicon predicted for the corresponding SSR primer, and the y-axis indicates the expected product size (bp). Multiple bands indicate multi-locus amplification.

When the *in silico* PCR results obtained from other genomes were examined, it was observed that some primers produced amplification profiles in certain genomes that differed from those obtained in the AHAU_CSS_1 genome, yielding specific amplicons with product sizes distinct from the expected ones (Figure 8, Figure 9, Figure 10, Figure 11 and Figure 12).

For example, primer AE096, which produced four distinct amplicons ranging between 185, 217, 260, and 1069 bp in the AHAU_CSS_1 genome, exhibited a similar amplification profile in the *Camellia sinensis* L618 genome (Figure 8), as well as in the ASM2053679v1 (Figure 9), ASM1731120v1 (Figure 11), and ASM2053686v1 (Figure 12) genomes. In contrast, a single amplicon of 242 bp was observed in the IND_Tea_TV1 genome (Figure 10).

In Figure 7, Figure 8, Figure 9, Figure 10, Figure 11 and Figure 12, due to visual limitations, bands of some primers producing amplicons of similar sizes (e.g., AE160, AE377) appear as merged and thicker single bands. Therefore, detailed amplification results for each genome are provided in Supplementary File S13.

## 3. Discussion

In this study, the genomic distribution, density, and structural characteristics of SSRs developed based on homologous genes, protein families, and conserved protein domains associated with defense responses and disease resistance were systematically evaluated. These targets were selected based on previous literature reporting their involvement in defense responses and disease resistance mechanisms against pathogens and pest infestation in tea (*Camellia sinensis*), where they were shown to exhibit upregulation or high expression under specific disease conditions [3,9,29,31,32,33,34,35,36]. The results demonstrate that SSRs are not randomly distributed across the genome; rather, they are distinctly enriched within the pQTL windows of the targeted locus, exhibiting clear gene-specific and gene family-specific patterns.

In this study, the selected genes and protein families were analyzed by identifying SSR motifs and markers based on their homologous loci in the AHAU_CSS_1 genome, which was used as the reference genome. This approach is consistent with methods previously employed for intergenomic gene comparisons within a species [47,48]. Within this framework, the present study addresses defense responses and disease resistance genes reported in different tea cultivars in the literature using a comparative genomics strategy based on their projection onto a single reference genome (AHAU_CSS_1).

Anthracnose is a severe disease caused by pathogens such as *Colletotrichum fructicola* and *Colletotrichum camelliae*, and is characterized by leaf lesions and tissue necrosis. *CsERF105*, which encodes a transcription factor belonging to the APETALA2/Ethylene Responsive Factor (AP2/ERF) family, has been identified as a positive regulator of resistance against anthracnose caused by *C. camelliae*. Suppression of this gene has been shown to result in loss of resistance and an increase in disease symptoms in the plant [31]. In this study, a total of six SSR primers were designed for the homologous locus of *CsERF105*, including six locus-specific and four polymorphic primers. This result is consistent with previous findings indicating that SSRs are frequently present within the genomic architecture of genes belonging to the AP2/ERF family. Indeed, large-scale comparative analyses spanning coding sequences have reported that AP2 domains represent the most highly enriched Pfam term among SSR-containing genes, followed by MYB and TCP transcription factors [47].

This observation suggests that transcription factors belonging to the AP2/ERF family frequently harbor intragenic SSRs, and that variation in these SSRs may have functional consequences in the regulation of stress responses and defense mechanisms [47,48,49]. The SSR enrichment observed at the homologous locus of *CsERF105* in AHAU_CSS_1, together with the polymorphic markers developed in this study, when considered alongside the well-established regulatory role of this gene [49,50], indicates a functional marker potential for monitoring anthracnose resistance at the molecular level.

*CsOPR3*, which has been shown to trigger resistance against another *Colletotrichum species*, *Colletotrichum fructicola*, functions in the biosynthesis of jasmonic acid (JA). It has been demonstrated that *CsOPR3* expression increases following pathogen attack, leading to elevated JA levels and, consequently, enhanced plant resistance [9]. In another study, *CsOPR3* was cloned and characterized in *Camellia sinensis*. *CsOPR3* was shown to be expressed in multiple organs and to be strongly induced by insect regurgitant and exogenous JA application during *Ectropis obliqua* infestation, indicating that this gene is directly involved in defense signaling pathways [51]. In the present study, although only a single SSR primer with a PIC value below 0.500 was obtained for *CsOPR3*, the observation that the same allele (334 bp) was conserved in two of the analyzed genomes, while a different allele (332 bp) was detected in a third genome, demonstrates the presence of genuine allelic variation at the homologous *CsOPR3* locus in the AHAU_CSS_1 genome. This finding is noteworthy, given the central biological role of *CsOPR3* in defense mechanisms in tea. The fact that *CsOPR3*, which participates in JA biosynthesis and is strongly induced under pathogen and herbivore attack, exhibits a more limited yet statistically informative SSR architecture suggests that this gene performs a highly conserved regulatory function. In this context, the primer derived from the *CsOPR3* homologous locus can be considered a targeted and functionally relevant marker candidate for monitoring JA-mediated defense responses.

*Exobasidium vexans* is an economically important and widespread fungal pathogen that primarily infects young leaves of the tea plant and causes the disease known as blister blight. In a study conducted by Jayaswall et al. [32], a total of 17 *CsPR-1* genes were identified in the tea genome, among which *CsPR-1-2*, *-4*, *-6*, *-7*, *-8*, *-9*, *-10*, *-14*, *-15*, and *-17* were shown to be markedly expressed during *E. vexans* infection, thereby activating resistance responses. These genes were reported to be associated with the salicylic acid (SA) and jasmonic acid (JA) signaling pathways; moreover, overexpression of specific *PR1* genes has been demonstrated to enhance resistance against fungal and oomycete pathogens and, in many cases, to strengthen tolerance to abiotic stresses as well [33,52,53,54,55].

In this study, among the 23 SSR primers developed for *PR1* genes located at different loci, four primers in particular exhibited products ranging from three to five alleles and displayed PIC values above 0.500, indicating the presence of meaningful polymorphism at specific *PR1* loci involved in *E. vexans*-associated defense responses. This finding suggests that these SSRs may serve not only as gene-specific markers but also as informative and selective candidates for monitoring resistance-related genetic variation.

R genes such as *RPM1* and *RPS2* have been reported to be highly expressed in resistant genotypes during the later stages of disease progression, particularly during the sporulation phase. These genes are involved in the recognition of pathogen effectors and play a critical role in restricting pathogen spread by triggering programmed cell death in infected cells through the initiation of the hypersensitive response [32,56]. Among the nine SSR primers obtained from loci associated with these genes, five exhibited two or more alleles, revealing high genetic variation within these loci and highlighting allelic differences that are thought to influence resistance responses through interactions of RPS2 with other host factors [57,58].

In another study conducted under *E. vexans* infection, transcription factors belonging to the WRKY and NAC (NAM) families were shown to regulate immune responses by controlling the expression of defense–resistance-related genes, while leucine-rich repeats (LRR) and proteins of the PRX and CHI families carrying specific conserved amino acid motifs were found to be highly upregulated in disease-tolerant genotypes and to possess structural roles in defense functions [34,59,60]. In particular, NBS-LRR (NLR) genes constitute the largest class of R genes and confer race-specific resistance against fungi, bacteria, oomycetes, viruses, and insects. These genes are widely used in breeding programs and marker-assisted selection (MAS) processes [61,62,63].

In the present study, 278 of the SSR primers were developed from genes associated with *Exobasidium vexans* and from protein families harboring conserved amino acid motifs, including WRKY, PRX, NAC, LRR, and CHI. This observation is consistent with the widespread occurrence and economic importance of *E. vexans*-associated diseases in tea, as well as with the extensive investigation of defense mechanisms against this pathogen in the literature. At the same time, this pattern reflects the presence of multiple suitable SSR loci within regions identified through amino acid motif–based proteome screening of these protein families. Moreover, two SSRs were identified in association with *Colletotrichum fructicola*, for which the involvement of the *CsOPR3* in resistance was previously functionally demonstrated by Chen et al. [9]. This distribution is consistent with expectations, considering that resistance genes reported in the literature are predominantly concentrated in *Exobasidium*-related diseases and that certain pathogen–host interactions have been relatively less explored. When locus and SSR primer numbers were evaluated together according to pathogen and pest species, the marker distribution was found to directly reflect the functional locus density defined in the literature.

Among pests, *Empoasca onukii* [35,36] and *Ectropis obliqua* [35] constitute another pest group that causes significant yield losses in tea cultivation and involves distinct molecular pathways in defense responses, allowing for the development of multiple SSR primers per locus in this study. In tea, infestation by *Ectropis obliqua* (tea geometrid), an important sap-feeding pest, has been shown to markedly upregulate the expression of genes involved in monoterpene and sesquiterpene biosynthesis, particularly *CsTPS10*, *CsTPS08*, *CsTPS30*, and *CsCHAT1*, as well as *CsOPR3*, *CsMAPK*, and *CsWRKY3* [35]. Similarly, defense and resistance against the green leafhopper *Empoasca onukii* has been shown to involve the activation of *CsBAHD93*, *CsBAHD94*, and *CsBAHD95* genes [36]. For these two pests, the large number of polymorphic (PIC > 0.500) and multi-allelic SSR primers (*n* = 21) obtained from homologous loci of the corresponding genes indicates that BAHD acyltransferases possess a variation-rich genomic architecture that is amenable to genotype-level differentiation within defense pathways responding to pest-derived stresses [64,65].

For another important pest, Empoasca vitis, six highly polymorphic (PIC > 0.600) and multi-allelic SSR primers were obtained relative to the number of loci investigated. In this context, increased expression of LOX, HPL1, CCoAOMT, CYP74B24, PR, and GLP genes, which have been reported to overlap with QTLs associated with disease resistance, particularly in cereals [66], as well as PKS-ER and allene oxide cyclase (AOC) genes, has been documented [29]. In the present study, although an SSR was identified for AOC, it could not be included in the SSR primer panel due to the inability to design a suitable primer.

In response to infestation by the pink tea mite, defense and resistance mechanisms have been shown to be centered on genes involved in the production of secondary metabolites, and the upregulation of these genes has been detected. These genes promote the accumulation of flavonoids and phenolic compounds that provide a chemical defense against the pest while activating genes involved in the flavonoid and phenylpropanoid biosynthesis pathways, such as PAL, C4H, 4CL, CHS, F3H, and FLS, during attacks by the tea mite (*Acaphylla theae*). Activation of these genes contributes to the plant defense response by supporting the synthesis of secondary metabolites with deterrent effects against pests, including catechins and lignin [3].

The SSR primers obtained for the mite pest *Acaphylla theae* (*n* = 22), particularly those derived from UGT94E5 and UGT91A1, were included in the SSR panel developed in this study due to their involvement in flavonoid pathways and their reported roles in the activation of defense-related compounds [67,68]. Within this panel, the identification of multi-allelic and highly polymorphic primers (PIC > 0.600) for UGT91A1 and RPM1 indicates that these genes exhibit a highly variable genomic architecture, suggestive of adaptive potential in pathogen- and pest-related defense responses.

The locus sets analyzed in this study, together with protein families containing specific amino acid motifs, exhibit a heterogeneous structure. Nevertheless, the fact that all gene groups could be addressed within the same analytical framework demonstrates that the applied pQTL-guided approach operates independently of locus architecture. In particular, the high SSR representation observed in multi-locus families such as WRKY, NAC, PRX, CHI, and LRR is a natural consequence of the amino acid motif-based proteome-wide screening strategy and the broad genomic distribution of these protein families. This indicates that SSR density does not arise from biologically irrelevant randomness but rather from a design strategy that is consistent with the targeted locus architecture.

The distribution of the motif types indicates that short-motif SSRs predominate in the defense–resistance-associated genomic regions prioritized in this study, whereas longer-motif SSRs exhibit a more limited but selective representation. When *Z*-scores calculated according to motif length were examined, a distinct yet balanced distribution was observed across different motif classes. The fact that tetranucleotide motifs exhibited the highest median values and *Z*-scores indicates that this motif group displays statistically stronger discriminative power and signal intensity. However, the maximum *Z*-scores were distributed over a broader range, particularly for di- and hexanucleotide motifs, suggesting the presence of a limited number of high-signal-producing SSRs within these motif classes. Overall, these results indicate that motif length alone is not a determining factor, while certain motif classes harbor SSRs with high discriminative potential. Taken together, the motif-length distribution of the SSRs identified in this study is consistent with a locus-centered selection design shaped by gene family architecture and functional context.

*Z*-scores calculated based on SSR densities and permutation-based threshold analyses clearly demonstrate that the pQTL hotspot signal is independent of SSR repeat number. The absence of a significant correlation between SSR repeat counts and *Z*-scores according to Spearman and Pearson correlation analyses indicates that hotspot signal strength cannot be reduced to simple microsatellite expansion; instead, it is associated with the genomic and functional context of the SSRs. This finding shows that the pQTL approach is capable of distinguishing context-sensitive density patterns rather than relying solely on quantitative SSR abundance.

When SSR repeat characteristics were evaluated across defense–resistance-related genes and protein families, the resulting distribution was found to directly reflect the multi-layered and targeted design strategy of the study. WRKY, PRX, NAC, CHI, and LRR, which belong to protein families identified through proteome-wide screening based on conserved amino acid motifs, were notable for exhibiting both the highest number of SSRs and closely similar mean repeat values. The high representation of SSRs at the corresponding loci of these protein families is attributable not to increased polymorphism, but rather to the deliberate identification of multiple loci through amino acid motif-based genome screening, followed by the design of individual SSRs for each locus. In these groups, mean repeat numbers generally falling within the 8–9 range indicate a balanced and consistent SSR architecture within families. In contrast, SSR motifs derived from other targeted genes represented by a single locus also exhibited relatively high mean repeat numbers (~7–11). Such high repeat numbers may indicate a strong SSR architecture, high conservation, and potential polymorphism associated with these loci. SSRs derived from these loci are therefore well-suited for prioritization within the defense–resistance framework. Overall, the SSR repeat profiles across genes and protein families are consistent with locus-centered selection based on amino acid motif-based screening and literature-supported functional targets.

The positional distribution of SSRs within the genome indicates that the majority of markers are concentrated in genic regions, particularly within introns, and in regulatory regions proximal to genes. In contrast, the very limited number of SSRs located in distal intergenic regions demonstrates that the screening was not conducted randomly across the genome but rather followed a targeted strategy constrained to the functional genomic environment of genes. It also demonstrates that the pQTL-based strategy adopted in this study provides a holistic framework capable of capturing both intragenic structural variation and regulatory variation with potential effects on gene expression. Moreover, the observed pattern is fully consistent with the SSR screening strategy, which was restricted to gene bodies and approximately ±5000 bp upstream and downstream regions of each gene, thereby confirming that the SSRs were selected from functionally meaningful regions within pQTLs. The presence of SSRs at comparable proportions on both strands across many genes and protein families indicates that the screening and design strategy was orientation-independent and genomically unbiased. Partial strand asymmetries observed in certain genes are associated with their local genomic context and sequence features, and are not of a magnitude sufficient to alter the overall distribution trend. Overall, SSRs exhibit a balanced distribution across both DNA strands, while mild strand-oriented asymmetries are observed in certain genes and gene families.

Normalized physical mapping demonstrates that SSRs are not only confined within pQTL boundaries but are also spatially distributed in a biologically meaningful manner across genic regions and gene-proximal regulatory areas. This observation directly supports that the SSR screening and design strategy employed in this study is not based on random genome-wide scanning, but rather on a pQTL-centered, functionally guided approach.

*In silico* PCR analyses revealed that the designed SSR primers can exhibit distinct yet specific amplification profiles across different tea genomes. The observation that the same primer yields single or multiple amplicons in different genomes reflects the structural diversity of tea genomes and differences in genomic architecture among cultivars. These results demonstrate that a substantial proportion of the designed SSR primers are applicable not only to the reference genome but also across multiple tea cultivars, indicating their potential utility as molecular tools for future genotyping, cultivar discrimination, and resistance-associated phenotypic analyses. Despite these in silico results, it is necessary to test the designed SSR primers under controlled laboratory conditions and to determine, at the population level, to what extent they reflect phenotypic discrimination along the defense/resistance axis against the targeted pathogens and pests. The relationship of these markers, selected with a pQTL focus, with the phenotype may vary depending on the genetic background of the population, linkage disequilibrium (LD), and recombination dynamics. Therefore, although the present study provides a comprehensive SSR resource supported by multi-genome-level in silico validation and biologically guided selection, population-based wet-laboratory validation studies are still required for these markers to be fully considered trait-diagnostic and to be routinely applied in breeding programs.

The machine learning approach was employed in this study not for exploratory purposes, but to prioritize SSR candidates confined to pQTL hotspot regions at the gene family level. The Random Forest-based model provided relative prioritization among SSRs that had passed statistical and genomic filtering steps, thereby refining the decision-making process, particularly for multi-locus gene families. In this context, the RF-based prioritization increased the efficiency of locus-level SSR selection by supporting the enrichment of hotspot-relevant candidates, particularly in multi-locus gene families. This approach positions machine learning not as a substitute for the biological and statistical framework, but as a complementary decision-support tool. Overall, this part of the study demonstrates the applicability of a pQTL-guided, statistically controlled, and, where appropriate, machine learning-assisted SSR marker development strategy based on homologous genes and gene families associated with defense responses and disease resistance.

## 4. Materials and Methods

### 4.1. Bioinformatics Tools and Packages

In this study, bioinformatic screening and data processing steps were carried out using R version 4.5.0 [69] within the RStudio environment version 2025.09.2+418 (Posit Software, PBC, Boston, MA, USA) [70]. Motif searches in protein sequences were performed using the Biostrings ::matchPattern function (Biostring package v2.76.0), while Biostrings::subseq was used for the identification of SSR regions. Gene and coding sequence (CDS) information was obtained from annotation files (genomic.gff), and protein motif analyses were conducted using the complete proteome FASTA file of *Camellia sinensis* cv. Shuchazao (GCF_004153795.1_AHAU_CSS_1_protein.faa). Data manipulation, grouping, summarization, permutation analyses, and *Z*-score-based comparisons were performed using the dplyr (version 1.1.4) and tidyr (version 1.3.2) R packages. To control randomness during permutation analyses, a fixed random seed (set.seed) was defined at the beginning of the analysis. In silico PCR analyses were conducted by targeting the genomic FASTA file of each genome using the Biostrings::matchPattern function.

### 4.2. Literature-Based Selection of Disease Resistance–Associated Genes

In this study, defense- and disease resistance-associated genes and protein families, related to various pathogens and pests in tea (*Camellia sinensis*) were selected based on previously published studies reporting their upregulation or high expression under specific disease conditions [3,9,29,31,32,33,34,35,36]. As cultivar-specific genome annotations were not available in some of these studies, the corresponding locus information was determined using the Tea Plant Information Archive (TPIA; https://tpia.teaplant.org/, accessed on 30 December 2025), based on the closest cultivars with available genome annotations reported in the reference studies. These loci were subsequently mapped onto the *Camellia sinensis* (L.) var. *sinensis* cv. Shuchazao reference genome used in this study (GenBank: GCA_004153795.1; AHAU_CSS_1 assembly) by BLAST-based sequence similarity analyses (using the NCBI BLAST web interface, https://blast.ncbi.nlm.nih.gov/Blast.cgi, accessed on 30 December 2025) to identify homologous loci. For genes for which locus information was not reported but expression primers were provided, the corresponding genomic loci were determined by aligning the primer sequences to the reference genome. This approach was employed to ensure that genes previously demonstrated to be associated with defense responses and disease resistance were evaluated within a unified and consistent genomic framework on the AHAU_CSS_1 genome for subsequent SSR identification and primer panel development.

### 4.3. Reference Genome Selection and Acquisition of Annotation Data

In this study, the AHAU_CSS_1 genome assembly of *Camellia sinensis* (L.) var. sinensis cv. Shuchazao, which is widely used in the literature, was selected as the curated reference genome (GenBank: GCA_004153795.1; RefSeq: GCF_004153795.1, NCBI). Genome sequences (FASTA), gene annotation files (GFF/GTF), and protein sequences (FAA) corresponding to the reference genome were obtained from the NCBI GenBank and RefSeq databases. All genomic coordinates, locus definitions, and downstream analyses were conducted based on the AHAU_CSS_1 assembly.

### 4.4. Mapping of SSR Motifs at Target Loci

In this study, SSR motifs were identified independently for each gene and protein, and permutation distributions and statistical threshold values were calculated on a locus-specific basis. For this purpose, a scanning window was generated by extending each locus region by ±5000 bp on both sides; window boundaries were trimmed to ensure that they did not exceed chromosome or contig lengths, and the corresponding genomic subsequences were extracted using the Biostrings::subseq function. SSR screening was performed on each window sequence after conversion to uppercase single-character strings, using a Perl-compatible regular expression approach. Accordingly, tandem repeats with motif lengths of 2–6 bp were searched, and minimum repeat thresholds were defined according to motif length as follows: ≥8 repeats for k = 2, ≥5 repeats for k = 3, ≥4 repeats for k = 4, and ≥3 repeats for k = 5–6 [71]. During SSR detection, consecutive repeat blocks were identified for each motif length, and SSR regions were defined as genomic segments in which motifs of a given length (k base pairs) occurred in tandem for at least the specified minimum number of repeats.

### 4.5. Identification of Putative QTL Hotspot Regions

#### 4.5.1. SSR Density-Based Detection of Putative QTL Hotspots

To characterize the genomic distribution patterns of SSRs and to identify regions of local enrichment around loci (putative QTL hotspots), a locus-centered sliding-window analysis was performed using the SSR coordinates defined in the previous step. For each target locus, the analysis region was defined as ±5000 bp upstream and downstream of the locus start and end coordinates. Within this region, windows of 1000 bp were applied using two offsets corresponding to a step size of 500 bp (0 and 500 bp), thereby generating overlapping windows. This strategy was adopted to reduce boundary-related artifacts and to capture local SSR density patterns with higher resolution. For each SSR record, it was evaluated whether the SSR start coordinate fell within the gene-specific analysis region, and the number of SSRs in the corresponding window was calculated according to the window index. For each gene, all defined windows (considering both offsets together) were included; windows containing no SSRs were filled with zero (0) values to generate a complete density matrix. The resulting SSR counts were normalized on a per-gene basis, and the mean (*µ*) and standard deviation (*σ*) were calculated for each locus. SSR density for each window was expressed using the *Z*-score [(*x* − *µ*)/*σ*]. In cases where the standard deviation was zero or undefined, the *Z*-score was not calculated. This rule and the parameters used (window size, step size, number of permutations) are clearly provided in the code shared in Supplementary File S14.

As a result of this analysis, subregions within the locus ±5 kb interval of each locus showing relative increases or decreases in SSR density were quantitatively defined using window-based *Z*-scores, and potential putative QTL hotspot (pQTL) regions were identified.

Following the locus-centered SSR density analysis, a permutation-based approach was applied to assess whether the observed SSR density patterns deviated from random expectations and to define statistical threshold values for locus-specific pQTL hotspot regions. The analysis was conducted for each gene using the SSR counts observed in 1000 bp windows across the previously defined gene ±5 kb region. For each locus, the total number of SSRs, as well as the mean (*µ*) and standard deviation (*σ*) of the observed window-based SSR counts, were calculated; loci with zero or undefined standard deviation or lacking SSRs were excluded from further analysis.

In the permutation analysis, 1000 independent permutations were performed for each locus by randomly redistributing the observed total number of SSRs within the locus-specific analysis region (gene ±5 kb), while preserving the total SSR count. In each permutation, randomly positioned SSRs were projected onto 1000 bp windows, SSR counts were calculated for each window, and *Z*-scores were derived using the locus-specific observed mean and standard deviation. For each permutation, the maximum *Z*-score (max-*Z*) observed across all windows within the locus region was recorded, thereby generating a permutation-based distribution of maximum SSR density values for each locus.

From the resulting permutation distribution, locus-specific statistical threshold values were defined. The maximum *Z*-scores corresponding to the moderate (95%) and high (99%) confidence levels were used as critical thresholds. Accordingly, windows in which the observed *Z*-scores exceeded the locus-specific 95% or 99% thresholds were considered candidate pQTL hotspots exhibiting statistically significant SSR enrichment. In cases where threshold exceedance was observed in consecutive windows, these windows were merged to define continuous hotspot regions, and for each region, the start and end coordinates, number of windows, and maximum *Z*-score are reported.

The relationship between SSR repeat number and QTL *Z*-scores was assessed using Spearman [72] and Pearson [73] correlation analyses.

#### 4.5.2. Selection and Prioritization of SSR Candidates Within pQTL Hotspot Regions

To identify candidate SSRs located within pQTL hotspot regions and to prioritize the strongest candidates for each locus, all SSR records obtained from the locus ±5 kb regions were evaluated using a permutation-based framework, with primary emphasis on the 99% permutation threshold. This choice was made to minimize false-positive hotspot identification arising from random density fluctuations and to retain regions with the highest level of statistical confidence. However, for some locus-specific regions with low SSR content, no significant hotspot window or SSR candidate was detected under the 99% threshold. To avoid the complete exclusion of such loci and to increase sensitivity in regions with rare or limited SSR content, a secondary threshold of 95% was also applied. This hierarchical threshold strategy provided a balanced trade-off between high specificity (99%) and inclusiveness/sensitivity (95%).

Selection of SSR candidates was restricted exclusively to SSRs that genomically overlapped with pQTL hotspot regions identified through permutation-based analysis. During the selection and prioritization of SSR candidates within pQTL hotspots, both repeat-based structural characteristics and locus context-dependent positional criteria were jointly considered. As the primary selection criterion, the repeat number of the SSR motif was used. Based on the assumption that SSRs with higher repeat numbers have higher mutation rates and, consequently, greater polymorphism potential, candidates were ranked in descending order according to repeat number. Among SSRs with identical repeat numbers, relative positional proximity to the locus was used as a secondary criterion, with preference given to SSRs located closer to the locus’s start or end coordinates. According to these criteria, SSRs located within pQTL hotspot regions were ranked for each locus based on repeat number and relative distance to the locus, and a maximum of 100 SSRs per locus were selected for progression to the subsequent analysis stage.

#### 4.5.3. Machine Learning-Assisted Prioritization of SSR Motifs Associated with Chitinase, LRR, NAC, WRKY, and Peroxidase Genes

To more comprehensively evaluate the association of SSRs belonging to the CHI, LRR, NAC, WRKY, and PRX [34] protein families with pQTL hotspot regions, and to prioritize candidate SSRs at the protein family level beyond locus-specific thresholds, a supervised machine learning-based classification approach was applied. In this analysis, all SSRs associated with the respective protein families were labeled as positive or negative classes, and a Random Forest–based model was used to estimate the probability of SSRs being associated with putative QTL hotspot regions. The positive class consisted of SSRs located within pQTL hotspot regions that exceeded the 99% significance threshold in locus-based permutation analyses. The negative class was defined as SSRs that were located within pQTL hotspot regions but did not exceed this significance threshold. The class label (positive/negative) was used as the target variable in the machine learning analysis. Datasets comprising SSR observations from the CHI, LRR, NAC, WRKY, and PRX families were randomly partitioned within each group into 70% training and 30% test subsets. The classification model was trained using the ranger package, which provides stable performance on high-dimensional datasets and imposes no level limitations on categorical variables. The model was constructed as a Random Forest consisting of 1000 trees, with the number of variables evaluated at each node set to three, and probability-based outputs were generated. Variable contributions to the model were assessed using a node-purity-based importance measure.

The trained model was applied to all SSRs belonging to the CHI, LRR, NAC, WRKY, and PRX families that had been selected based on conserved amino acid motifs, and for each SSR, the probability of association with pQTL hotspot regions was calculated. SSRs were ranked in descending order according to these probability values, and candidate SSRs with the highest relative priority at the gene family level were identified.

From the SSRs obtained through machine learning-based prioritization, an additional filtering and evaluation step was applied to select the most suitable candidates for experimental validation and final marker panel construction. At this stage, SSRs with a probability of belonging to the positive class of 0.7 or higher were considered high-confidence candidates. In addition, motif-length-dependent minimum repeat number criteria were applied; at least eight consecutive repeats were required for di-, tri-, and tetranucleotide motifs, and at least four consecutive repeats were required for penta- and hexanucleotide motifs. Relative distance to the locus was used as a supporting criterion for prioritizing candidates that passed the threshold.

As a result of this multi-criteria evaluation, the highest-priority SSRs were selected for each locus, and a final marker panel containing a maximum of five SSRs per locus was constructed. All SSRs included in the machine learning analysis in this study were derived from loci located within pQTL hotspot regions identified in previous steps. Accordingly, the machine learning approach was not used to select SSRs outside QTL regions, but rather to provide relative prioritization and supporting evidence among SSR candidates confined to pQTL hotspot regions.

#### 4.5.4. Random Forest Ablation Quantification; WRKY Example

To determine the effectiveness of the RF analysis, an ablation analysis was performed using the WRKY family as an example. For this purpose, all SSR candidates within the WRKY family with available RF scores (predicted probability of being hotspot-positive) were first labeled; candidates overlapping the 99% pQTL/hotspot SSR set were defined as hotspot-positive (positive class), whereas the remaining candidates were defined as hotspot-negative (negative class). Then, the same structural filter was applied to both selection strategies: SSRs with motif length 2–4 were included if the repeat number was ≥8, and SSRs with motif length 5–6 were included if the repeat number was ≥4. The two prioritization strategies were compared under the same selection rule (top-5 SSR candidates per locus; locus = GenBank × locus id). In the ML-based strategy, candidates were restricted to those with the predicted probability of being hotspot-positive ≥0.70 and, within each locus, were ranked by decreasing probability of being positive, followed by decreasing repeat number and increasing distance to the gene, and the top five candidates were selected. In the baseline (RF-off) strategy, candidates were ranked within each locus by decreasing repeat number and increasing distance to the gene, and the top five candidates were selected. For both strategies, enrichment was calculated as the proportion of hotspot-positive SSRs among the selected candidates (positive rate = number of positive/number of selected); this proportion was reported both at the locus level and overall across all selected candidates, and the uplift value was defined as the difference between the overall positive rate values of the ML-based and baseline strategies.

#### 4.5.5. Construction of Final SSR Marker Panels

For target loci other than CHI, LRR, NAC, WRKY, and PRX, the construction of the final SSR panel was initiated by evaluating SSR candidates identified within pQTL hotspot regions defined using the 99% permutation threshold. From these candidates, structural filtering based on motif length and the number of consecutive motif repeats was applied to select SSRs with high experimental applicability and polymorphism potential.

Accordingly, a minimum of eight consecutive repeats was required for SSRs with di-, tri-, and tetranucleotide motifs, while a minimum of four consecutive repeats was required for SSRs with penta- and hexanucleotide motifs [71]. SSRs that did not meet these thresholds were excluded from further analysis. Following filtering, the remaining SSRs were first ranked in descending order based on motif repeat number; among SSRs with identical repeat numbers, a secondary ranking was performed based on motif length and genomic start coordinates.

### 4.6. Primer Design for Identified SSR Markers

Primer design for the selected SSRs was performed using Primer3 v2.6.1 (Boston, MA, USA). based on the reference genome sequence. For each SSR locus, 200 bp flanking sequences upstream and downstream of the SSR region were extracted, and primer design was directed to target this interval. This strategy aimed to ensure that the designed primers would generate specific and experimentally applicable amplicons encompassing the SSR locus.

During primer design, key parameters such as primer length, melting temperature (Tm), GC content, and amplicon size were constrained within ranges commonly accepted in the literature. Primer length was set to 18–25 base pairs, Tm values were constrained to approximately 50–65 °C, and GC content was limited to 35–65%. The target amplicon length was restricted to 100–400 bp. A single primer pair was designed for each SSR locus, and primer quality was evaluated based on primer penalty scores and complementarity metrics reported by Primer3.

### 4.7. In Silico PCR

To evaluate the generalizability and genomic positional consistency of the primer pairs designed for the identified SSRs across different tea genotypes, five *Camellia sinensis* genome assemblies (Table 2), together with AHAU_CSS_1, were used for *in silico* PCR screening.

These genomes were selected to represent both *C. sinensis* var. *sinensis* (CSS) and var. *assamica* (CSA) cultivars; priority was given to assemblies generated at the chromosome level, with low contig numbers and high assembly continuity.

The *in silico* PCR approach was employed to enable comparative evaluation of SSR loci across different genomic backgrounds and to test the cultivar-level applicability of marker candidates. For this purpose, the genomic FASTA file of each genome was used as a template, and each primer pair was systematically screened across the entire genome using the Biostrings package.

During the *in silico* PCR simulation, no base mismatches were allowed for primer–genome alignments (maximum mismatch = 0), and potential amplicons generated by primer pairs were limited to a maximum length of 1000 bp. This ensured that only fully matching primer binding events and amplification products likely to occur under experimental PCR conditions were considered. For each primer pair, both forward primer–reverse primer reverse-complement and reverse primer–forward primer reverse-complement combinations were evaluated, thereby capturing all possible binding orientations across the genome. This strategy allowed simultaneous identification of single or multiple primer binding behaviors and the potential amplicon regions generated by each binding combination.

As a result of the analysis, for each primer pair, (i) the total number of binding sites detected across the genome, (ii) the number of potential amplicons, and (iii) the genomic coordinates of the amplicons were determined. The resulting data were compiled to enable comparative assessment of primer specificity and potential risks of multiple amplification events. Following the *in silico* PCR analyses, PIC values were calculated to evaluate the degree of polymorphism among the designed SSR primers in order to prioritize SSR primer candidates. In practical applications, primers with PIC values > 0.500 are generally considered to have high discriminative power [74].

### 4.8. Integrated Pipeline for pQTL-Guided and Machine Learning-Assisted SSR Marker Development

**Figure 13:**
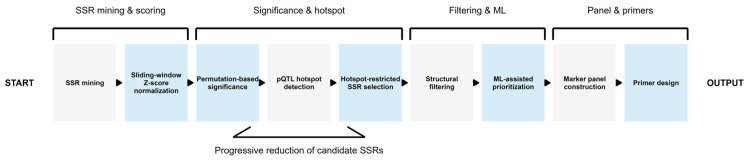
Schematic overview of the pQTL-guided and machine learning-assisted integrated workflow applied in this study for defense- and disease resistance-oriented SSR marker development.

When the analytical steps applied in this study are considered as a whole, an integrated and stepwise workflow emerges for the development of defense- and disease resistance-oriented SSR markers, guided by pQTL analysis and supported by machine learning. This workflow comprises biological function- or gene-specific SSR identification, density profiling, *Z*-score normalization, permutation-based significance testing, identification of pQTL hotspot regions, hotspot-restricted SSR selection, structural filtering, machine learning-assisted marker prioritization where suitable data are available, construction of the marker panel, and finally primer design. Rather than aiming to introduce an independent methodological framework, this approach represents a logical synthesis of the statistical, genomic, and machine learning-based filtering and prioritization steps described in detail in the preceding sections of the study. Through this process, the candidate marker space was progressively narrowed, resulting in a biologically meaningful and experimentally applicable SSR marker panel (Figure 13).

All key parameters, thresholds, and Random Forest settings used in the workflow are summarized in Table 4.

## 5. Conclusions

The pathogen- and pest-specific SSR panels developed in this study, targeting defense- and resistance-associated genes and protein families, constitute a structurally defined resource with strong biological context for experimental validation and downstream genotyping applications. The developed SSR panels were designed based on genes and protein families that have been associated with defense–resistance mechanisms through transcriptomic, expression-based, and functionally supported evidence in the literature and were subjected to multilayered statistical filtering steps. The primers, developed from homologous loci based on a single reference genome (AHAU_CSS_1), are consistent with standard practices in SSR-based analyses. Their laboratory performance (in vitro amplification efficiency and specificity), as well as their transferability across diverse tea populations, will be evaluated in subsequent studies, thereby establishing their transferability to marker-assisted selection (MAS) and defense–resistance-oriented applications. To the best of our knowledge, this work represents the first comprehensive and integrative SSR primer panel developed in tea that is explicitly focused on pathogen and pest-oriented defense and resistance mechanisms.

## Figures and Tables

**Table 1:** Defense- and resistance-associated genes and protein families used for SSR development in this study.

Gene, Protein Family	Biological Function/Role (*)	Reference
*CsOPR3*	12-oxophytodienoate reductase; jasmonic acid biosynthesis pathway	[9]
*RPP13*	RPM1 gene	[3]
*RF45*	RPM1 gene	[3]
*R1A-4*	RPM1 gene	[3]
*RPM1*	RPM1 gene	[3]
*UGT91A1*	Flavonoid precursor	[3]
*UGT94ES*	Flavonoid precursor	[3]
*At4g26220*	Flavonoid precursor	[3]
*At1g67980*	Flavonoid precursor	[3]
PBL7	A0A4S4DNL6	[29]
GLP	A0A4S4EHC7	[29]
LOX	A0A4S4EI95	[29]
CcoAOMT	A0A4V3WNP8	[29]
PKS-ER	A0A4V3WQ16	[29]
CYP74B24	A0A4S4E216	[29]
*CsERF105*	A nuclear-localized Ethylene-responsive transcription factor	[31]
*RPS2*	Resistance protein (NBS-LRR); pathogen recognition and defense activation	[32]
BEAT	BAHD acyltransferase	[32]
PR1	Pathogenesis-related protein 1; marker for systemic acquired resistance	[33]
STS14	PR1	[33]
Chitinase	Hydrolyzes chitin in fungal cell walls; key enzyme in plant defense	[34]
Peroxsidase	Involved in reactive oxygen species detoxification and pathogen defense	[34]
NAC	Transcription factor regulating stress responses and development	[34]
LRR	Leucine-rich repeat domain involved in protein–protein interactions, commonly in resistance proteins	[34]
WRKY	Transcription factor family regulating pathogen and abiotic stress responses	[34]
EBOS	Terpene synthase	[35]
GDS	Terpene synthase	[35]
*MAPK17*	Mitogen-activated protein kinase; signal transduction in stress and defense pathways	[35]
AQUA21	Aquaporin	[35]
*PIP2.2*	Aquaporin	[35]
*JAZ1*	Jasmonate-zim-domain protein	[35]
CPRX	Cationic peroxidase	[35]
*ERF8*	CsERF	[35]
AMAT	BAHD acyltransferase	[36]
HHT	BAHD acyltransferase	[36]
BAHD-AT	BAHD acyltransferase	[36]
HCT	BAHD acyltransferase	[36]
BAHD-DCR	BAHD acyltransferase	[36]
FACT	BAHD acyltransferase	[36]
ACT	BAHD acyltransferase	[36]
VS	BAHD acyltransferase	[36]

* Information on the biological functions of the genes and protein families was obtained from the studies reporting the respective genes.

**Table 2:** Assemblies and GenBank accession numbers of tea plant genomes used in this study.

Assembly	GenBank	Scientific Name	Cultivar
AHAU_CSS_1	GCA_004153795.1	*Camellia sinensis*	Shuchazao
ASM1731120v1	GCA_017311205.1	*Camellia sinensis* var. *sinensis*	Tieguanyin
IND_Tea_TV1	GCA_028456175.1	*Camellia sinensis* var. *assamica*	TV1
ASM2053679v1	GCA_020536795.1	*Camellia sinensis* var. *assamica*	TES-34
*Camellia sinensis* L618 reference annotation	GCA_963931755.2	*Camellia sinensis*	
ASM2053686v1	GCA_020536865.1	*Camellia sinensis* var. *assamica*	UPASI-3

**Table 3:** Distribution of SSR primer numbers across pathogen and pest species.

Pathogen and Pest	Number of Loci	Number of Markers	Single-Product Markers	Multi-Product Markes
*Colletotrichum fructicola*	1	1	0	1
*Empoasca onukii*	12	33	9	24
*Empoasca vitis*	6	15	6	9
*Exobasidium vexans*	154	278	105	173
*Acaphylla theae*	8	22	6	16
*Ectropis obliqua*	13	31	14	17
*Colletotrichum camelliae*	1	6	2	4

**Table 4:** Key analysis parameters and thresholds used in the workflow.

Parameter	Setting (Value)
Analysis region (locus ± flanking)	±5000 bp
Motif lengths	2–6 bp
Minimum repeat threshold	k = 2:≥8; k = 3:≥5; k = 4:≥4; k = 5–6:≥3
Sliding window size	1000 bp
Window step size	500 bp (offsets: 0 and 500 bp)
Number of permutations	1000
Hotspot significance threshold	95% and 99%; final panel: 99%
Handling of σ = 0 cases	*Z*-score set to NA; *Z* not calculated; excluded from hotspot calling
Random Forest settings	ranger; 70/30 train-test split; 1000 trees; mtry = 3; probability = TRUE; importance = node purity; high-confidence cutoff: prob_pos ≥ 0.70
Primer length	18–20 bp
Tm	50–65 °C
GC ratio	35–65%
Amplicon size	100–400 bp
Primer flanking region	±200 bp
Mismatch (in silico PCR)	0
Max amplicon (in silico PCR):	1000 bp

## Data Availability

Data are contained within the article and Supplementary Materials.

## Supplementary Materials

The following supporting information can be downloaded at: https://www.mdpi.com/article/10.3390/plants15030454/s1, Supplementary File S1: Final SSR catalogue; Supplementary File S2: Final target amino acid motif-based loci; Supplementary File S3 Proteome-wide amino acid motif atlas; Supplementary File S4. Initial SSR screening output; Supplementary File S5: Summary statistics, repeat distributions, repeats per gene, SSR motif length per gene and protein family, distance metrics, correlation analyses, SSR counts per pathogen/pest and SSR per pQT. Supplementary File S6: Strand orientation, distance stats, genic and intergenic SSR distribution and SSR per pQTL coordinates; Supplementary File S7: Detailed physical maps of SSR and gene positions across pQTL regions for all scaffolds; Supplementary File S8: Random Forest ablation analysis results; Supplementary File S9: Final SSR primer dataset; Supplementary File S10: SSR primer locus per pathogen and pest, and summary of unique SSR primers by gene and protein family; Supplementary File S11: Multi-genome in silico PCR outputs; Supplementary File S12: SSR primers’ PIC analyses; Supplementary File S13: High PIC SSR primers (PIC > 0.500); Supplementary File S14: R code for Z-score calculation of SSR density, including handling of σ = 0/undefined cases.

## References

[1] Jia X., Zhang W., Fernie A.R., Wen W. (2021). *Camellia sinensis* (Tea). Trends Genet..

[2] Tanaka J., Taniguchi F., Kole C. (2007). Tea. Technical Crops. Genome Mapping and Molecular Breeding in Plants.

[3] Chen L., Shu Z., Zhou D., Zhou H., Wang J., Feng Y., Zheng S., He W. (2024). Metabolite Profiling and Transcriptome Analyses Reveal Defense Regulatory Network Against Pink Tea Mite Invasion in Tea Plant. BMC Genom..

[4] Lehmann-Danzinger H. (2000). Diseases and Pests of Tea: Overview and Possibilities of Integrated Pest and Disease Management. J. Agric. Trop. Subtrop..

[5] Sharma G., Majumder S., Ghosh A., Bhattacharya M. (2024). Metabolomic Responses of Tea [*Camellia sinensis* (L.) O. Kuntze] Leaves to Red Spider Mite [*Oligonychus coffeae* (Nietner)] and Tea Mosquito Bug [*Helopeltis theivora* Waterhouse] Infestation: A GC–MS-Based Study. Bull. Natl. Res. Cent..

[6] Zhao X., Chen S., Wang S., Shan W., Wang X., Lin Y., Su F., Yang Z., Yu X. (2020). Defensive Responses of Tea Plants (*Camellia sinensis*) Against Tea Green Leafhopper Attack: A Multi-Omics Study. Front. Plant Sci..

[7] Pandey A.K., Sinniah G.D., Babu A., Tanti A. (2021). How the Global Tea Industry Copes with Fungal Diseases—Challenges and Opportunities. Plant Dis..

[8] Roy S., Barooah A.K., Ahmed K.Z., Baruah R.D., Prasad A.K., Mukhopadhyay A. (2020). Impact of Climate Change on Tea Pest Status in Northeast India and Effective Plans for Mitigation. Acta Ecol. Sin..

[9] Chen S., Zhang L., Cai X., Li X., Bian L., Luo Z., Li Z., Chen Z., Xin Z. (2020). *(E)-Nerolidol* Is a Volatile Signal That Induces Defenses Against Insects and Pathogens in Tea Plants. Hortic. Res..

[10] Fan J., Zhang X., Jiang W., Xu J., Wu M., Dai X., Xu F., Niu S., He Y. (2025). Integrative Transcriptome and Metabolome Analysis Uncovers the *Toxoptera aurantii* (Hemiptera: Aphididae) Response of Two *Camellia sinensis* (Ericales: *Theaceae*) Cultivars. J. Econ. Entomol..

[11] Liu Y., Yan Y., Ma L., Cao D., Jin X. (2024). Physiological and Metabolomic Analyses Reveal the Resistance Response Mechanism to Tea Aphid Infestation in New Shoots of Tea Plants (*Camellia sinensis*). Plant Stress.

[12] Naskar S., Roy C., Ghosh S., Mukhopadhyay A., Hazarika L.K., Chaudhuri R.K., Roy S., Chakraborti D. (2021). Elicitation of Biomolecules as Host Defense Arsenals During Insect Attacks on Tea Plants (*Camellia sinensis* (L.) Kuntze). Appl. Microbiol. Biotechnol..

[13] Sarmah S.R., Bhattacharyya P.N., Barooah A.K. (2020). Microbial Biocides—Viable Alternatives to Chemicals for Tea Disease Management. J. Biol. Control.

[14] Wang W., Zhou X., Hu Q., Wang Q., Zhou Y., Yu J., Ge S., Zhang L., Guo H., Tang M. (2025). Lignin Metabolism Is Crucial in the Plant Responses to *Tambocerus elongatus* (Shen) in *Camellia sinensis* L.. Plants.

[15] Zhang J., Yu Y., Qian X., Zhang X., Li X., Sun X. (2024). Recent Advances in the Specialized Metabolites Mediating Resistance to Insect Pests and Pathogens in Tea Plants (*Camellia sinensis*). Plants.

[16] Hu Y., Zhang M., Lu M., Wu Y., Jing T., Zhao M., Zhao Y., Feng Y., Wang J., Gao T. (2022). Salicylic Acid Carboxyl Glucosyltransferase UGT87E7 Regulates Disease Resistance in *Camellia sinensis*. Plant Physiol..

[17] Hajiboland R. (2017). Environmental and Nutritional Requirements for Tea Cultivation. Folia Hortic..

[18] Kaczyński P., Iwaniuk P., Jankowska M., Orywal K., Socha K., Perkowski M., Farhan J.A., Łozowicka B. (2024). Pesticide residues in common and herbal teas combined with risk assessment and transfer to the infusion. Chemosphere.

[19] Sen S., Rai M., Das D., Chandra S., Acharya K. (2020). Blister blight: A threatened problem in tea industry—A review. J. King Saud Univ. Sci..

[20] Zheng R., Zhan J., Liu L., Ma Y., Wang Z., Xie L., He D. (2019). Factors and minimal subsidy associated with tea farmers’ willingness to adopt ecological pest management. Sustainability.

[21] Ding X., Lu Q., Li L., Li H., Sarkar A. (2023). Measuring the impact of relative deprivation on tea farmers’ pesticide application behavior: The case of Shaanxi, Sichuan, Zhejiang, and Anhui Province, China. Horticulturae.

[22] Xie S., Feng H., Yang F., Zhao Z., Hu X., Wei C., Liang T., Li H., Geng Y. (2019). Does dual reduction in chemical fertilizer and pesticides improve nutrient loss and tea yield and quality? A pilot study in a green tea garden in Shaoxing, Zhejiang Province, China. Environ. Sci. Pollut. Res..

[23] Yatoo A.M., Ali M.N., Baba Z.A., Hassan B. (2021). Sustainable management of diseases and pests in crops by vermicompost and vermicompost tea: A review. Agron. Sustain. Dev..

[24] Chen Z.-M., Sun X.-L., Dong W.-X., Chen L., Apostolides Z., Chen Z.-M. (2012). Genetics and chemistry of the resistance of tea plant to pests. Global Tea Breeding.

[25] Mores A., Borrelli G.M., Laidò G., Petruzzino G., Pecchioni N., Amoroso L.G.M., Desiderio F., Mazzucotelli E., Mastrangelo A.M., Marone D. (2021). Genomic approaches to identify molecular bases of crop resistance to diseases and to develop future breeding strategies. Int. J. Mol. Sci..

[26] Paul M., Mahla J.S., Upadhyay D.K., Das D., Wankhade M., Kumar M., Lallawmkimi M.C. (2025). Integration of genetic resistance mechanisms in sustainable crop breeding programs—A review. J. Adv. Biol. Biotechnol..

[27] Jiang Q., Guo N., Wang S., Bai J., Yu Y., Liu S. (2025). Identification of causal agent of gray blight disease in *Camellia sinensis* and screening of resistance cultivars. J. Exp. Bot..

[28] Nisha S.N., Prabu G., Mandal A.K.A. (2018). Biochemical and molecular studies on the resistance mechanisms in tea [*Camellia sinensis* (L.) O. Kuntze] against blister blight disease. Physiol. Mol. Biol. Plants.

[29] Wang F., Zhang B., Wen D., Liu R., Yao X., Chen Z., Mu R., Pei H., Liu M., Song B. (2022). Chromosome-scale genome assembly of *Camellia sinensis* combined with multi-omics provides insights into its responses to infestation with green leafhoppers. Front. Plant Sci..

[30] Xu L.-Y., Su J.-J., Zhang C.-K., Hao M., Zhou Z.-W., Chen X.-H., Zheng S.-Z. (2025). Identification of key genes associated with anthracnose resistance in *Camellia sinensis*. PLoS ONE.

[31] Zhang C., Li H., Mei P., Ye Y., Liu D., Gong Y., Liu H., Yao M., Ma C. (2025). QTL detection and candidate gene analysis of the anthracnose resistance locus in tea plant (*Camellia sinensis*). J. Integr. Agric..

[32] Jayaswall K., Mahajan P., Singh G., Parmar R., Seth R., Raina A., Swarnkar M.K., Singh A.K., Shankar R., Sharma R.K. (2016). Transcriptome analysis reveals candidate genes involved in blister blight defense in tea (*Camellia sinensis* (L.) Kuntze). Sci. Rep..

[33] Zhang Q., Guo N., Zhang Y., Yu Y., Liu S. (2022). Genome-wide characterization and expression analysis of pathogenesis-related 1 (PR-1) gene family in tea plant (*Camellia sinensis* (L.) O. Kuntze) in response to blister-blight disease stress. Int. J. Mol. Sci..

[34] Singh G., Singh G., Seth R., Parmar R., Singh P., Singh V., Kumar S., Sharma R.K. (2019). Functional annotation and characterization of hypothetical protein involved in blister blight tolerance in tea (*Camellia sinensis* (L.) O. Kuntze). J. Plant Biochem. Biotechnol..

[35] Qiao D., Yang C., Guo Y., Chen J., Chen Z. (2023). Transcriptome and co-expression network analysis uncover the key genes mediated by endogenous defense hormones in tea plant in response to the infestation of *Empoasca onukii* Matsuda. Beverage Plant Res..

[36] Qiao D., Yang C., Mi X., Tang M., Liang S., Chen Z. (2024). Genome-wide identification of tea plant (*Camellia sinensis*) BAHD acyltransferases reveals their role in response to herbivorous pests. BMC Plant Biol..

[37] Geethanjali S., Kadirvel P., Anumalla M., Hemanth Sadhana N., Annamalai A., Ali J. (2024). Streamlining of simple sequence repeat data mining methodologies and pipelines for crop scanning. Plants.

[38] Mason A.S., Mason A.S. (2015). SSR genotyping. Plant Genotyping: Methods and Protocols.

[39] Tillault A., Yevtushenko D.P. (2019). Simple sequence repeat analysis of new potato varieties developed in Alberta, Canada. Plant Direct.

[40] Zhao M., Shu G., Hu Y., Cao G., Wang Y. (2023). Pattern and variation in simple sequence repeat (SSR) at different genomic regions and its implications to maize evolution and breeding. BMC Genom..

[41] Batashova M., Kryvoruchko L., Makaova-Melamud B., Tyshchenko V., Spanoghe M. (2024). Application of SSR markers for assessment of genetic similarity and genotype identification in local winter wheat breeding program. Stud. Biol..

[42] Zhao C., Qiu J., Agarwal G., Wang J., Ren X., Xia H., Guo B., Ma C., Wan S., Bertioli D.J. (2017). Genome-wide discovery of microsatellite markers from diploid progenitor species, *Arachis duranensis* and *A. ipaensis*, and their application in cultivated peanut (*A. hypogaea*). Front. Plant Sci..

[43] Sari D., Sari H., Ikten C., Toker C. (2023). Genome-wide discovery of di-nucleotide SSR markers based on whole genome re-sequencing data of *Cicer arietinum* L. and *Cicer reticulatum* Ladiz. Sci. Rep..

[44] Zhao T., Miao L., Zou M., Hussain I., Yu H., Li J., Sun N., Kong L., Wang S., Li J. (2024). Development of SSRs based on the whole genome and screening of bolting-resistant SSR marker in *Brassica oleracea* L.. Horticulturae.

[45] Zuki Z.M., Rafii M.Y., Ramli A., Oladosu Y., Latif M.A., Sijam K., Ismail M.R., Sarif H.M. (2020). Segregation analysis for bacterial leaf blight disease resistance genes in rice ‘MR219′ using SSR marker. Chil. J. Agric. Res..

[46] Abbasi Holasou H., Panahi B., Shahi A., Nami Y. (2024). Integration of machine learning models with microsatellite markers: New avenue in world grapevine germplasm characterization. Biochem. Biophys. Rep..

[47] Song X., Yang Q., Bai Y., Gong K., Wu T., Yu T., Pei Q., Duan W., Huang Z., Wang Z. (2021). Comprehensive analysis of SSRs and database construction using all complete gene-coding sequences in major horticultural and representative plants. Hortic. Res..

[48] Sureshkumar S., Chhabra A., Guo Y., Balasubramanian S. (2025). Simple sequence repeats and their expansions: Role in plant development, environmental response and adaptation. New Phytol..

[49] Portis E., Lanteri S., Barchi L., Portis F., Valente L., Toppino L., Rotino G.L., Acquadro A. (2018). Comprehensive characterization of simple sequence repeats in eggplant (*Solanum melongena* L.) genome and construction of a web resource. Front. Plant Sci..

[50] Ahmadi A.J., Ahmadikhah A. (2022). Occurrence of simple sequence repeats in cDNA sequences of safflower (*Carthamus tinctorius*) reveals the importance of SSR-containing genes for cell biology and dynamic response to environmental cues. Front. Plant Sci..

[51] Xin Z., Zhang J., Ge L., Lei S., Han J., Zhang X., Li X., Sun X. (2017). A putative 12-oxophytodienoate reductase gene *CsOPR3* from *Camellia sinensis* is involved in wound and herbivore infestation responses. Gene.

[52] Liu F., Xi L., Fu N. (2025). Genome-wide development of simple sequence repeat (SSR) markers at 2-Mb intervals in lotus (*Nelumbo* Adans.). BMC Genom..

[53] Wang P., Zhou J., Sun W., Li H., Li D., Zhuge Q. (2023). Characteristics and function of the pathogenesis-related protein 1 gene family in poplar. Plant Sci..

[54] Li J., Luo C., Yang X., Peng L., Lu T., Yang J., Zhang X., Xie Y., Yang Z., Xu F. (2023). Genome-wide identification of the mango pathogenesis-related 1 (PR1) gene family and functional analysis of *MiPR1A* genes in transgenic *Arabidopsis*. Sci. Hortic..

[55] Javed T., Wang W., Sun T., Shen L., Feng X., Huang J., Zhang S. (2024). Pathogenesis-related 1 (PR1) protein family genes involved in sugarcane responses to *Ustilago scitaminea* stress. Int. J. Mol. Sci..

[56] Meteignier L.-V., el Oirdi M., Cohen M., Barff T., Matteau D., Lucier J.-F., Rodrigue S., Jacques P.-E., Yoshioka K., Moffett P. (2017). Translatome analysis of an NB-LRR immune response identifies important contributors to plant immunity in *Arabidopsis*. J. Exp. Bot..

[57] Caicedo A.L., Schaal B.A., Kunkel B.N. (1999). Diversity and molecular evolution of the *RPS2* resistance gene in *Arabidopsis thaliana*. Proc. Natl. Acad. Sci. USA.

[58] Banerjee D., Zhang X., Bent A.F. (2001). The leucine-rich repeat domain can determine effective interaction between *RPS2* and other host factors in *Arabidopsis RPS2*-mediated disease resistance. Genetics.

[59] Wang W., Cao H., Wang J., Zhang H. (2025). Recent advances in functional assays of WRKY transcription factors in plant immunity against pathogens. Front. Plant Sci..

[60] Viswanath K.K., Kuo S.-Y., Tu C.-W., Hsu Y.-H., Huang Y.-W., Hu C.-C. (2023). The role of plant transcription factors in the fight against plant viruses. Int. J. Mol. Sci..

[61] Bae S.-H., Zoclanclounon Y.A.B., Park G.-H., Lee J.-D., Kim T.-H. (2025). Genome-wide in silico analysis of leucine-rich repeat R-genes in *Perilla citriodora*: Classification and expression insights. Genes.

[62] Marone D., Russo M., Laidò G., De Leonardis A., Mastrangelo A. (2013). Plant nucleotide binding site–leucine-rich repeat (NBS-LRR) genes: Active guardians in host defense responses. Int. J. Mol. Sci..

[63] Yue J., Meyers B.C., Chen J., Tian D., Yang S. (2012). Tracing the origin and evolutionary history of plant nucleotide-binding site–leucine-rich repeat (NBS-LRR) genes. New Phytol..

[64] Kruse L.H., Fehr B., Chobirko J.D., Moghe G.D. (2023). Phylogenomic analyses across land plants reveals motifs and coexpression patterns useful for functional prediction in the BAHD acyltransferase family. Front. Plant Sci..

[65] Xu D., Wang Z., Zhuang W., Wang T., Xie Y. (2023). Family characteristics, phylogenetic reconstruction, and potential applications of the plant BAHD acyltransferase family. Front. Plant Sci..

[66] Biniaz Y., Tahmasebi A., Tahmasebi A., Albrectsen B.R., Poczai P., Afsharifar A. (2022). Transcriptome meta-analysis identifies candidate hub genes and pathways of pathogen stress responses in *Arabidopsis thaliana*. Biology.

[67] Gharabli H., Della Gala V., Welner D.H. (2023). The function of UDP-glycosyltransferases in plants and their possible use in crop protection. Biotechnol. Adv..

[68] Rehman H.M., Nawaz M.A., Shah Z.H., Ludwig-Müller J., Chung G., Ahmad M.Q., Yang S.H., Lee S.I. (2018). Comparative genomic and transcriptomic analyses of family-1 UDP glycosyltransferase in three *Brassica* species and *Arabidopsis* indicates stress-responsive regulation. Sci. Rep..

[69] R Core Team (2025). R: A Language and Environment for Statistical Computing. R Foundation for Statistical Computing, Vienna, Austria. https://www.R-project.org/.

[70] Posit Team (2025). RStudio: Integrated Development Environment for R; Posit Software.

[71] Ma J.-Q., Yao M.-Z., Ma C.-L., Wang X.-C., Jin J.-Q., Wang X.-M., Chen L. (2014). Construction of a SSR-based genetic map and identification of QTLs for catechins content in tea plant (*Camellia sinensis*). PLoS ONE.

[72] Spearman C. (1904). The proof and measurement of association between two things. Am. J. Psychol..

[73] Cohen J., Cohen P., West S.G., Aiken L.S. (2013). Applied Multiple Regression/Correlation Analysis for the Behavioral Sciences.

[74] Eminoğlu A., İzmirli Ş.G., Beriş F.Ş., Dinçer D., Yazıcı K. (2025). SSR genotyping of 200 tea (*Camellia sinensis*) clones obtained by selection and DNA barcoding of 12 varietal registration candidates. BMC Plant Biol..

