# Supplementary figures and images for: Identification and Development of Pathogen- and Pest-Specific Defense–Resistance-Associated SSR Marker Candidates Assisted by Machine Learning and Discovery of Putative QTL Hotspots in Camellia sinensis
Source: Plants (Basel). 2026 Feb 2;15(3):454. doi: 10.3390/plants15030454 (PMC12899447; doi:10.3390/plants15030454)

1048

1060

1078

1085

1093

1104

1125

1141

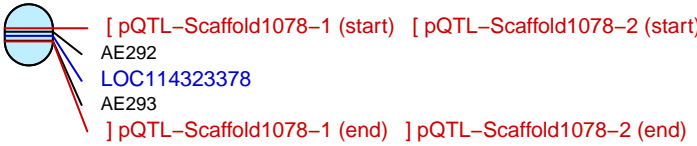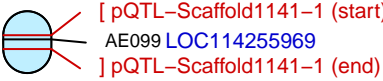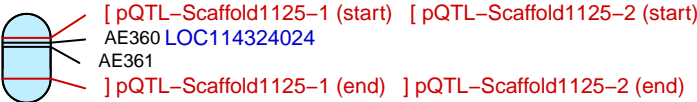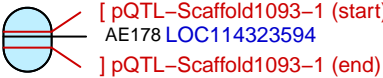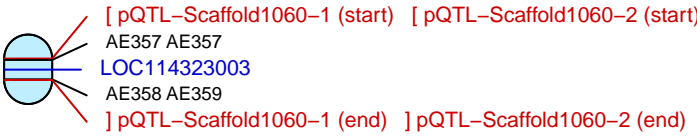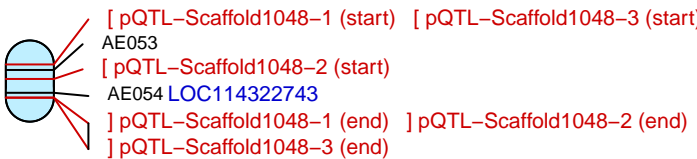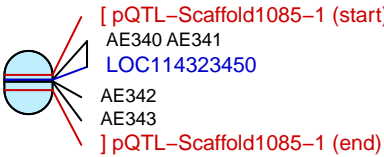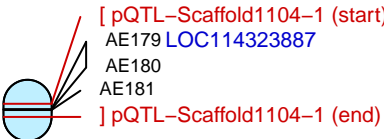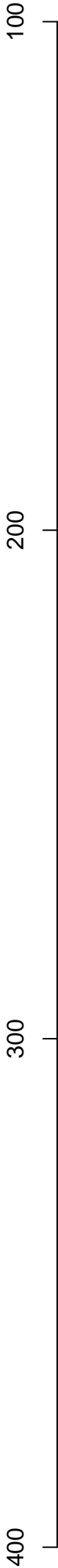

11425

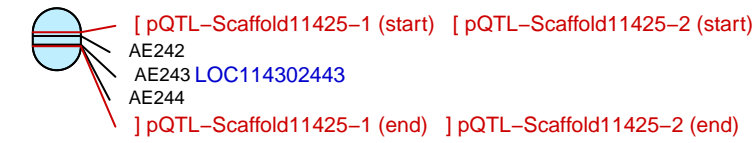

1163

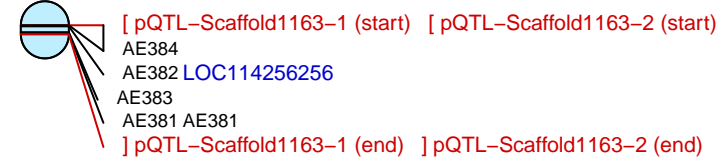

1167

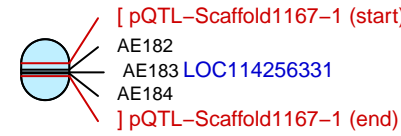

1170

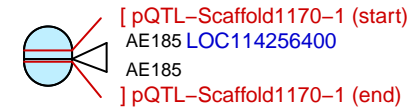

1175

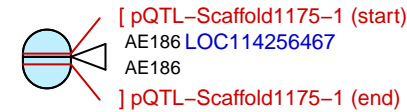

1182

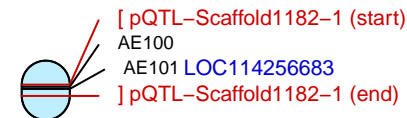

1197

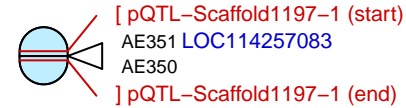

1199

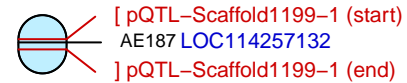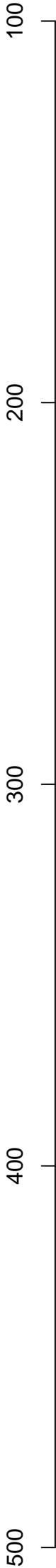

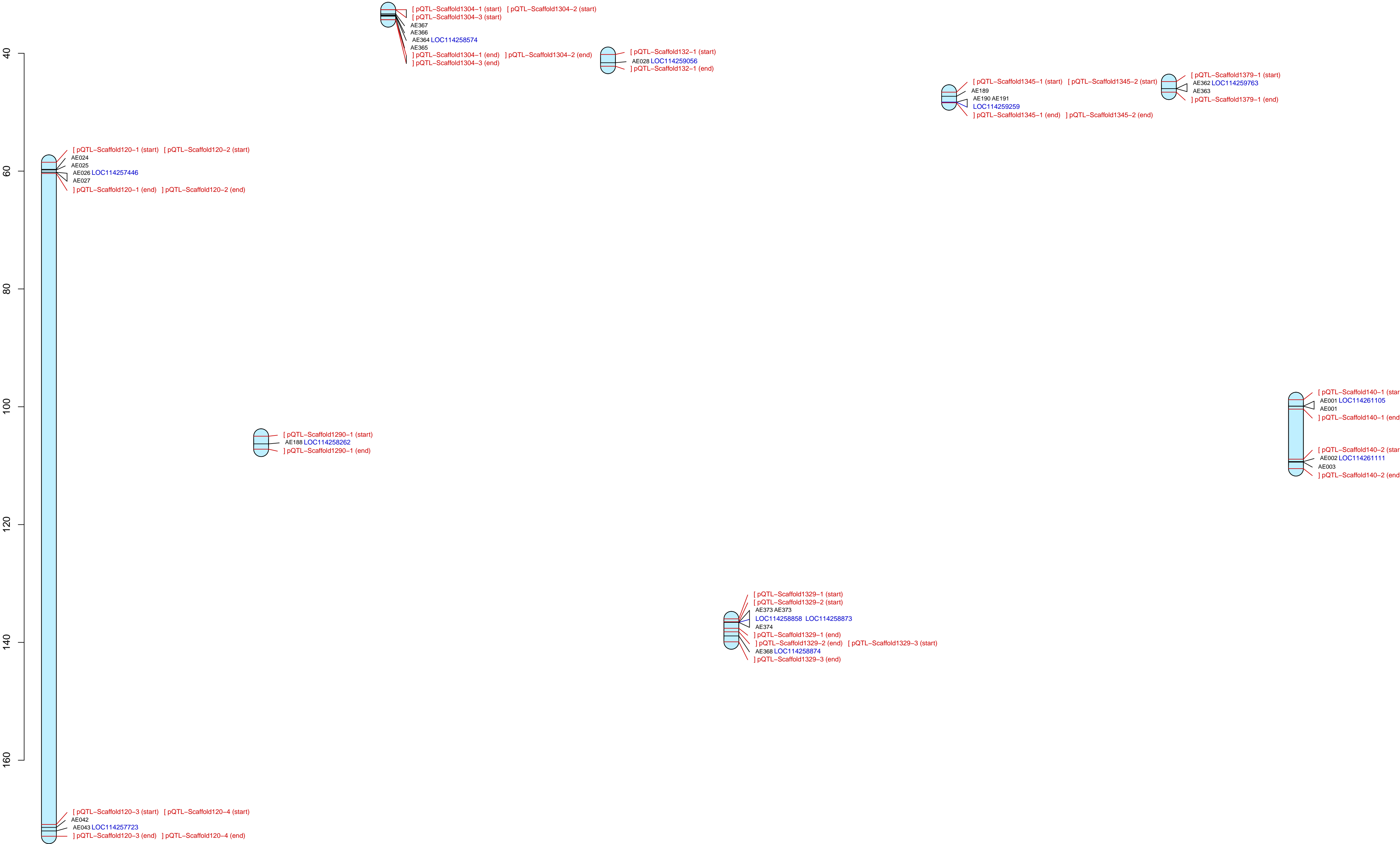

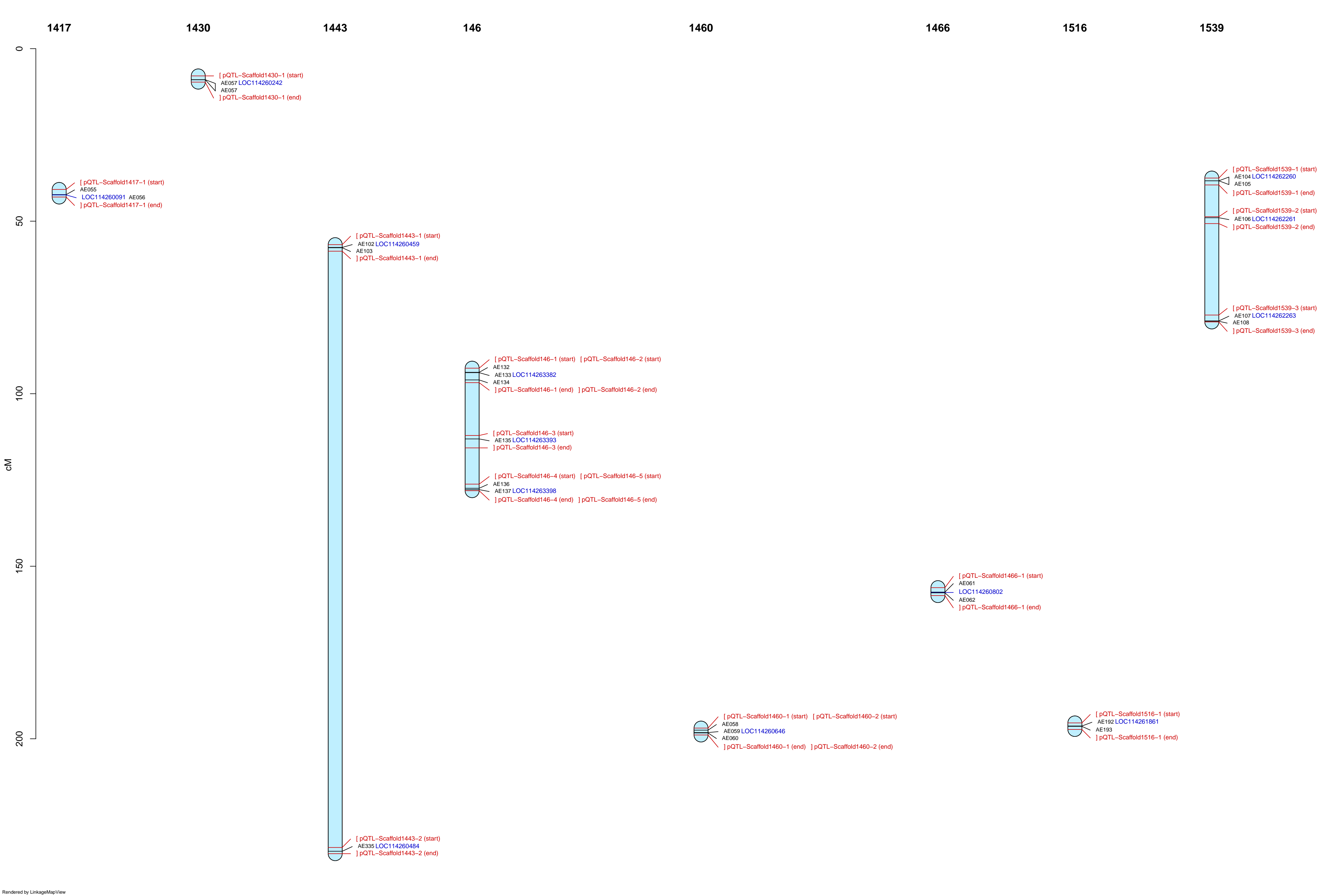

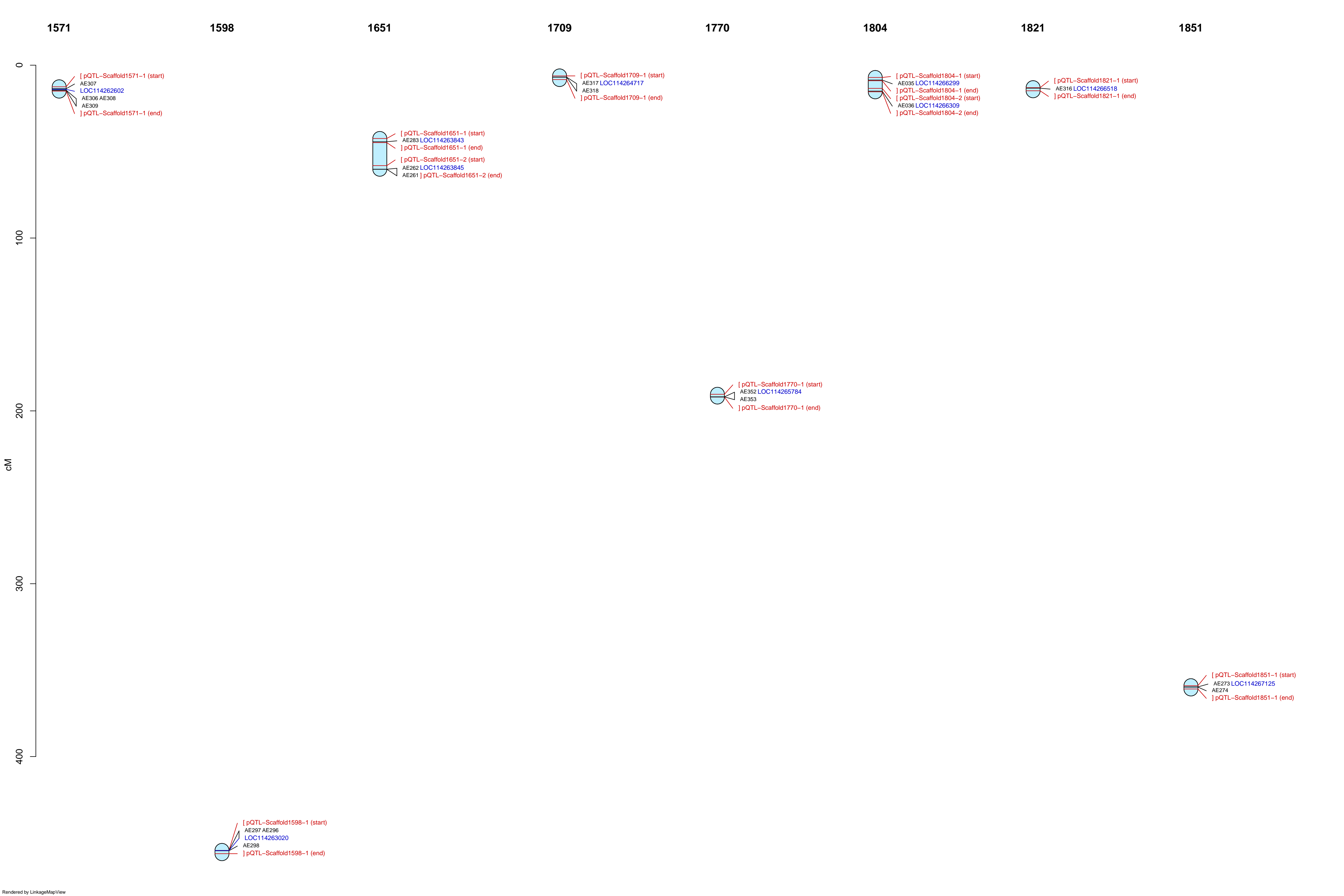

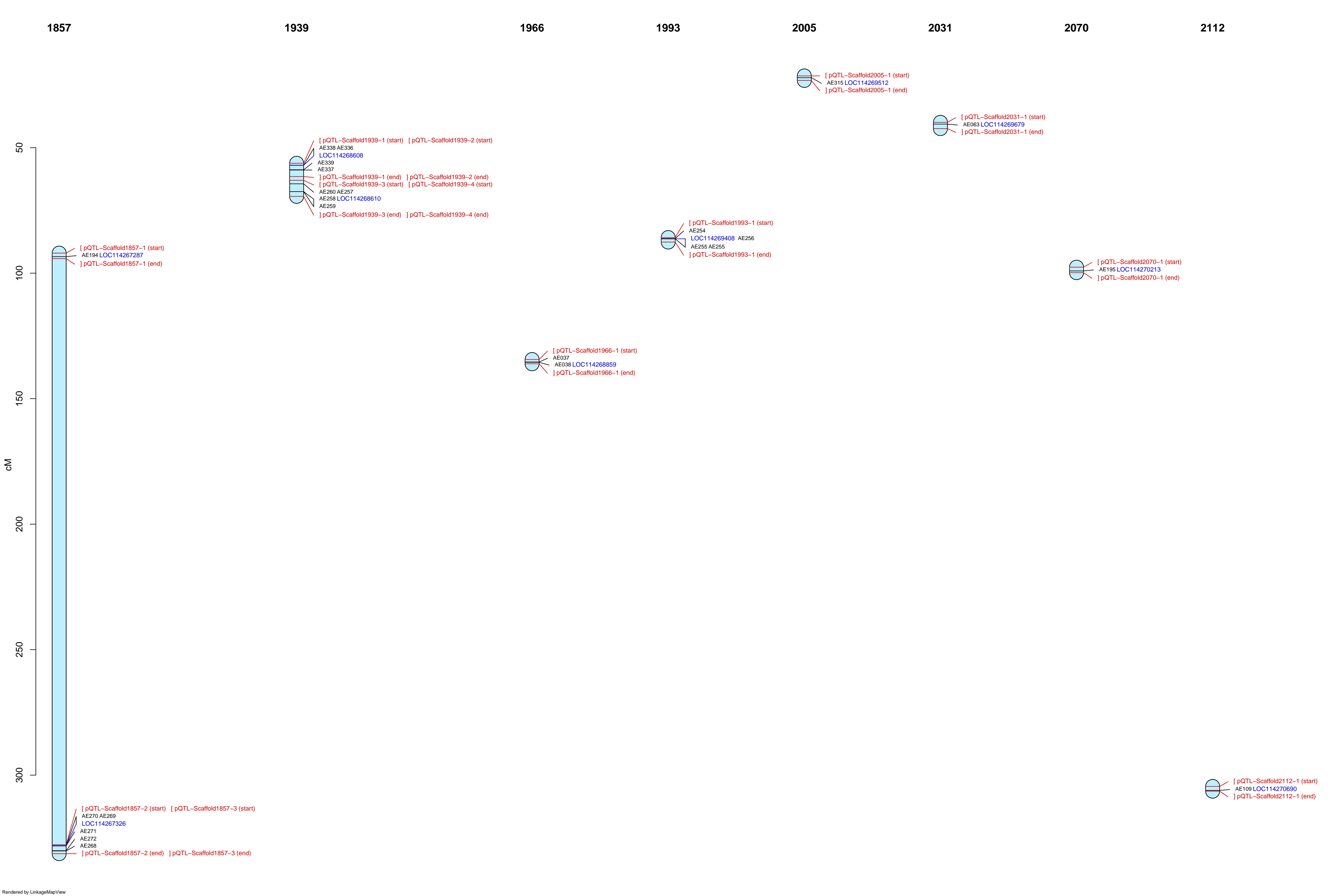

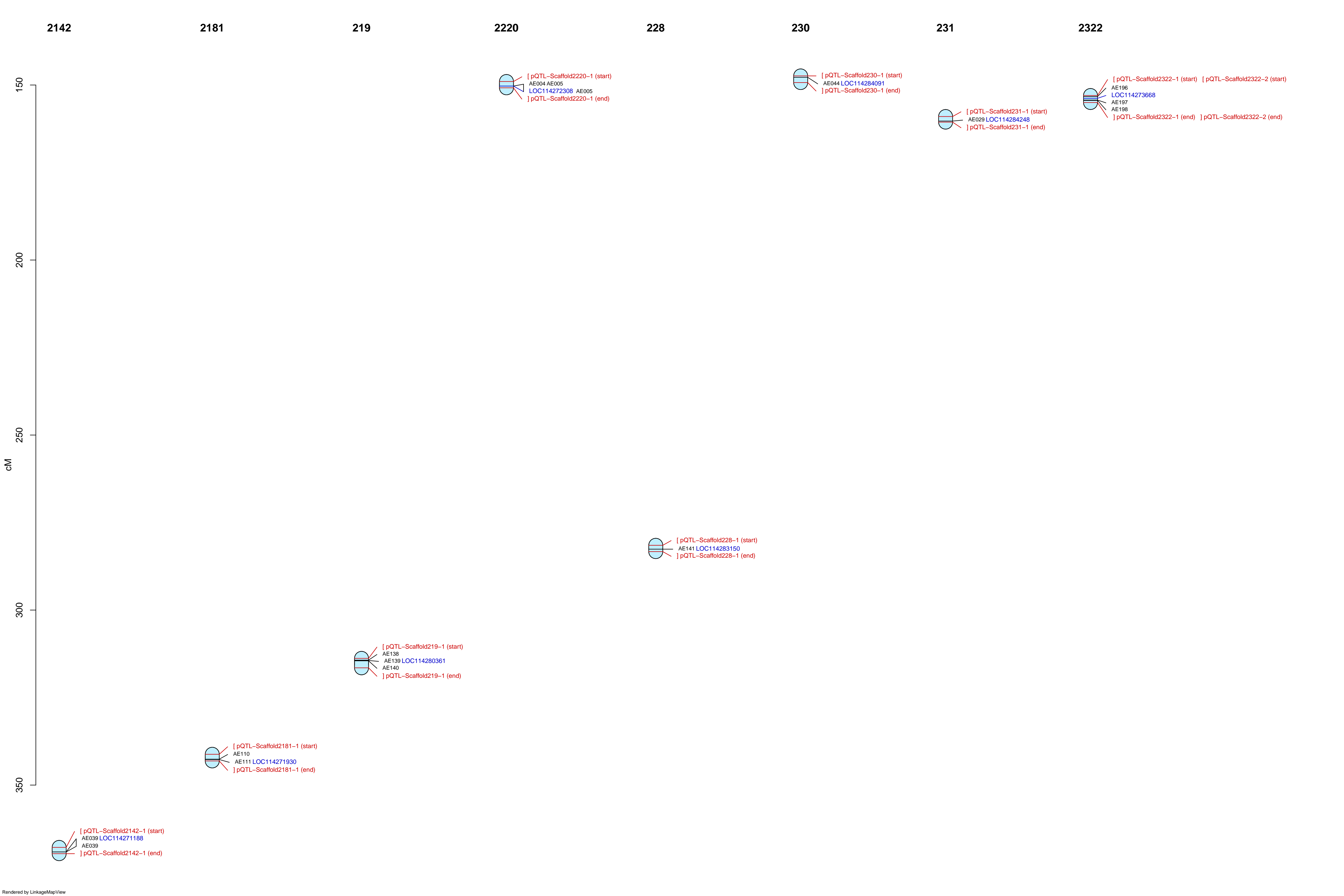

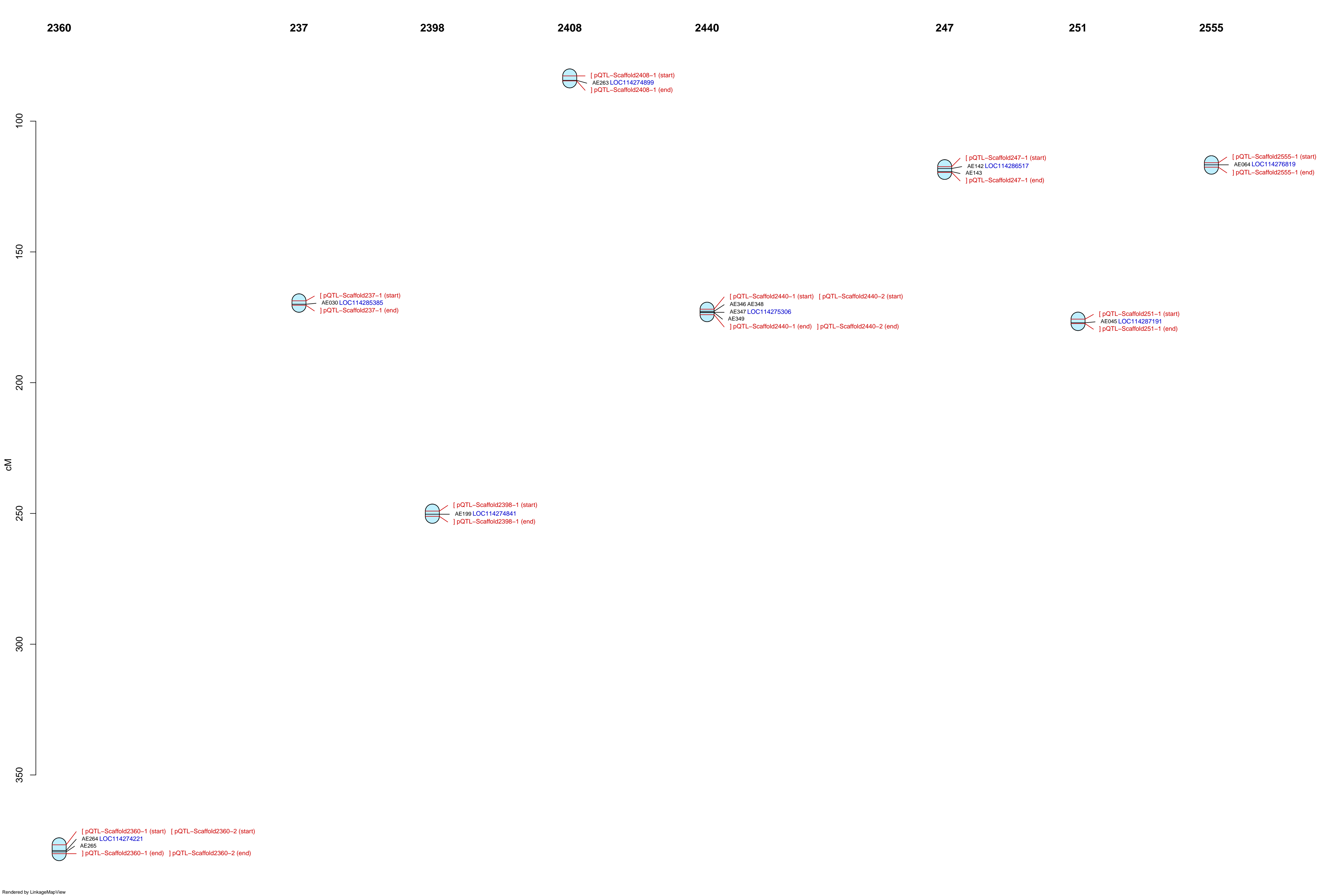

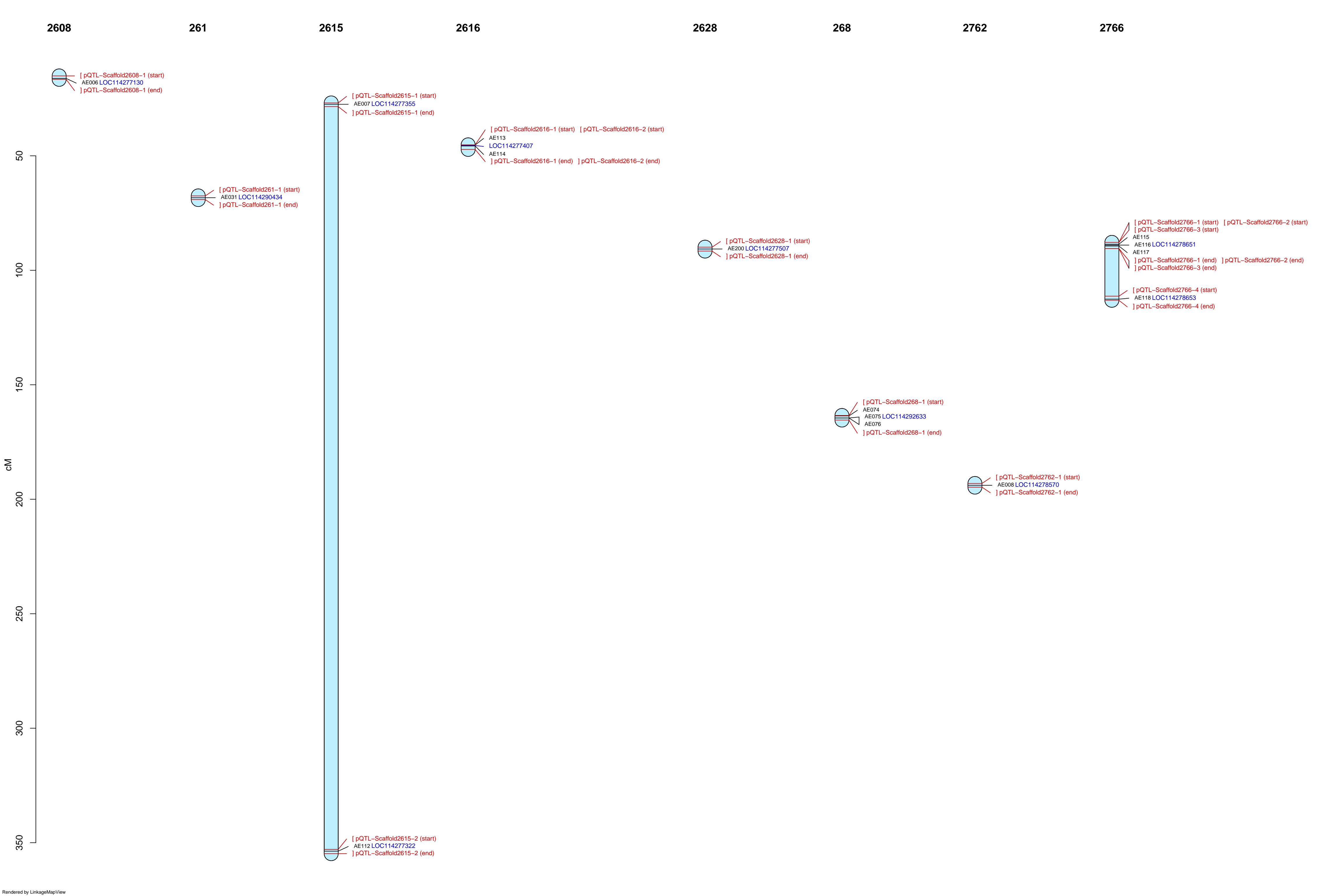

2776

2838

2840

2859

287

2881

293

2936

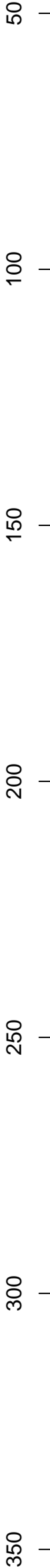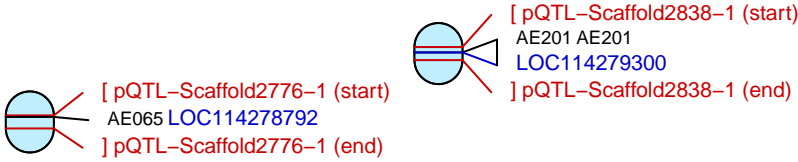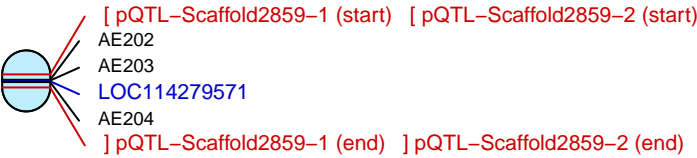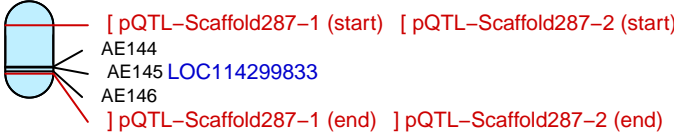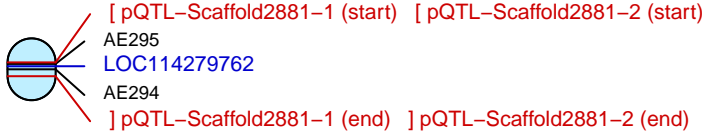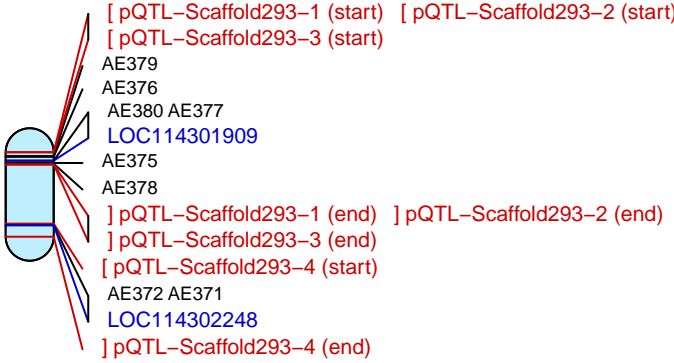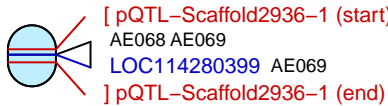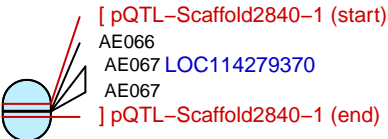

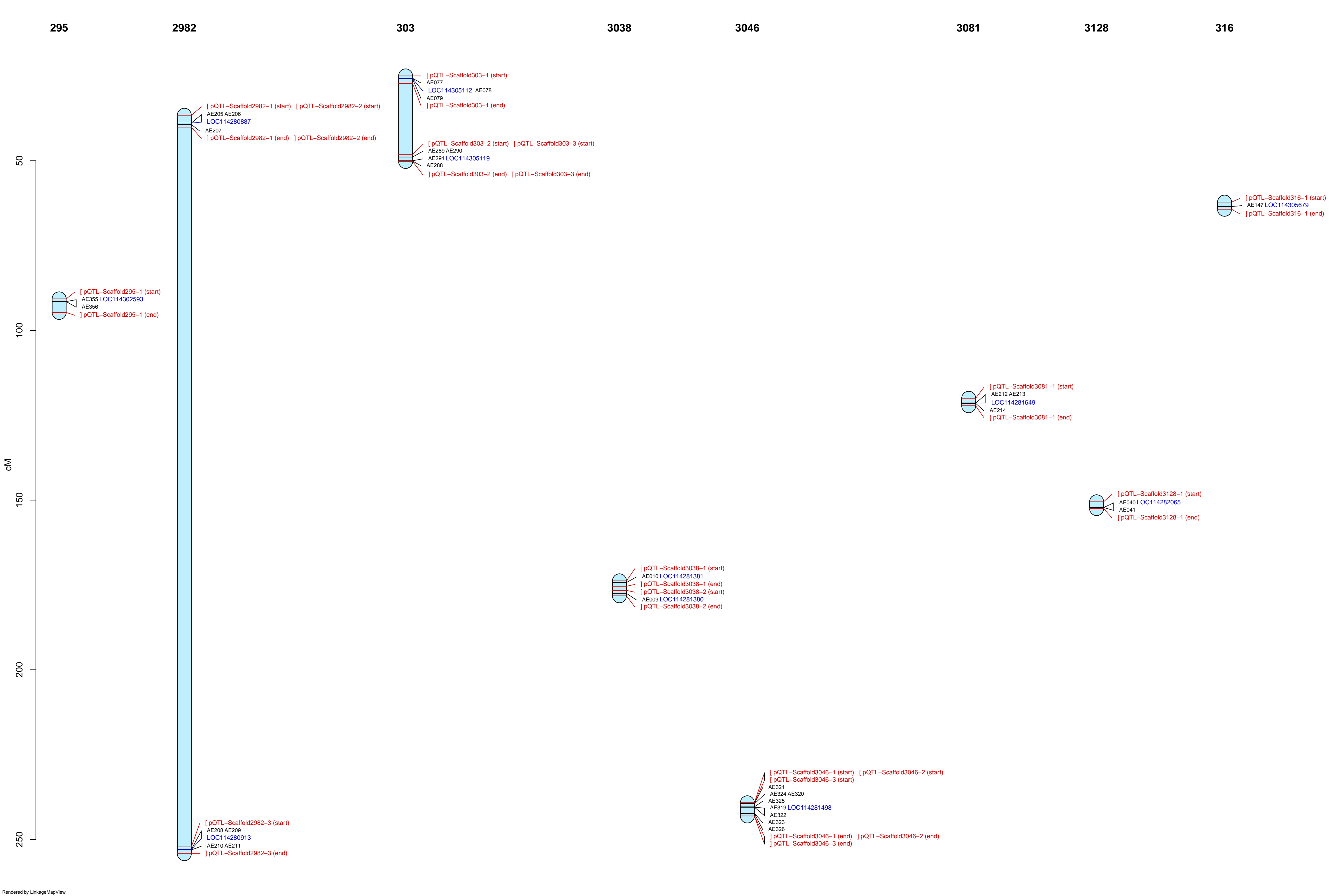

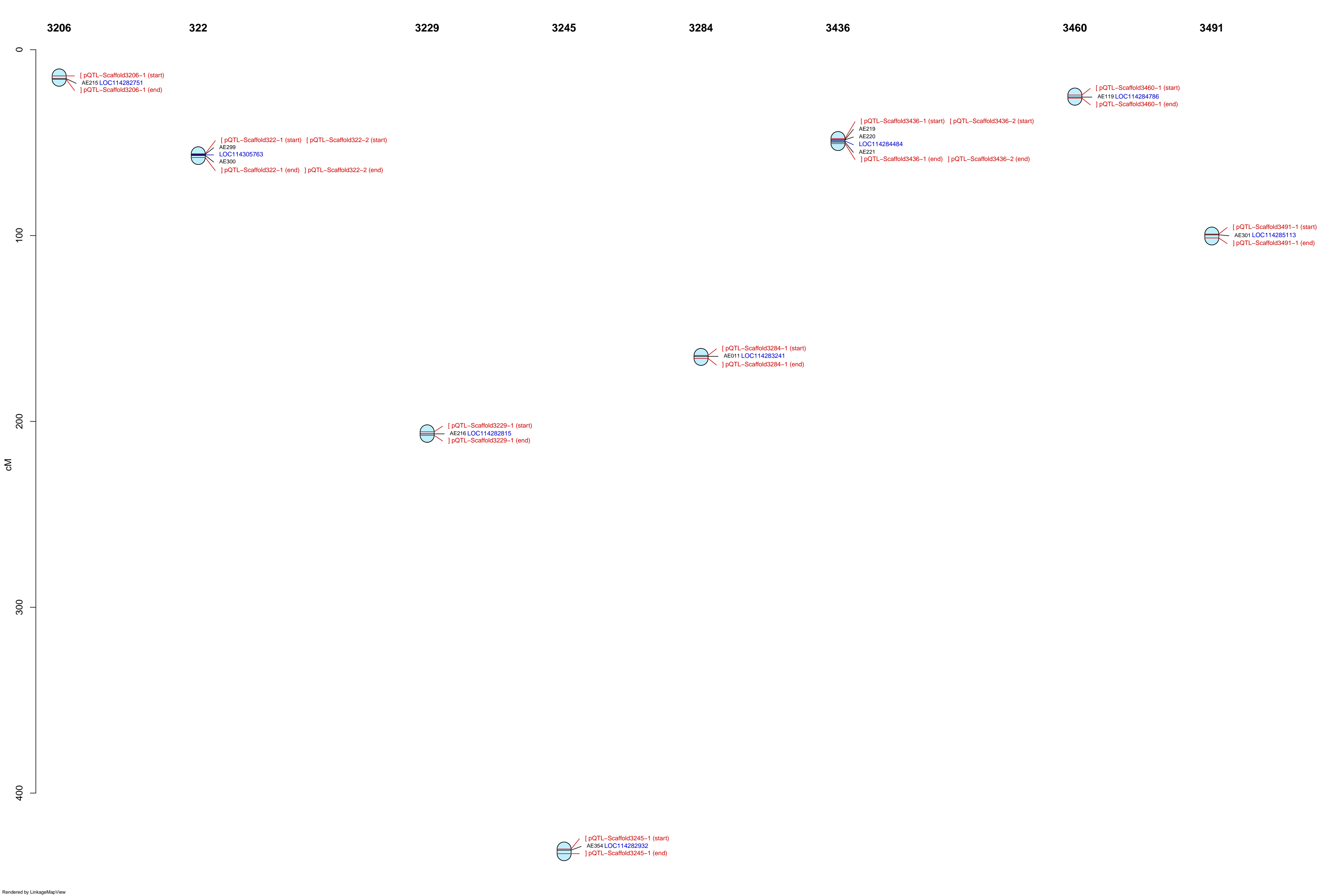

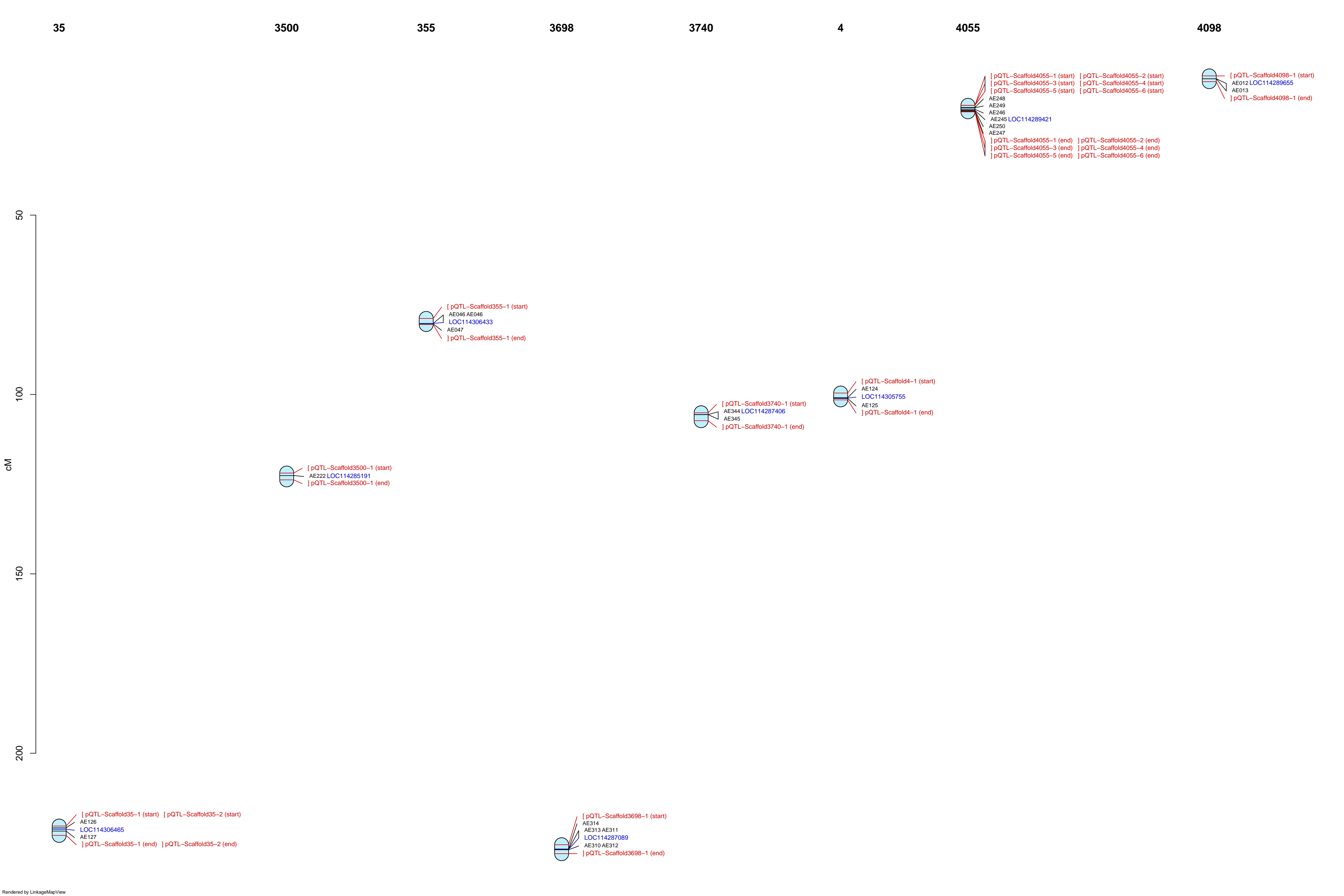

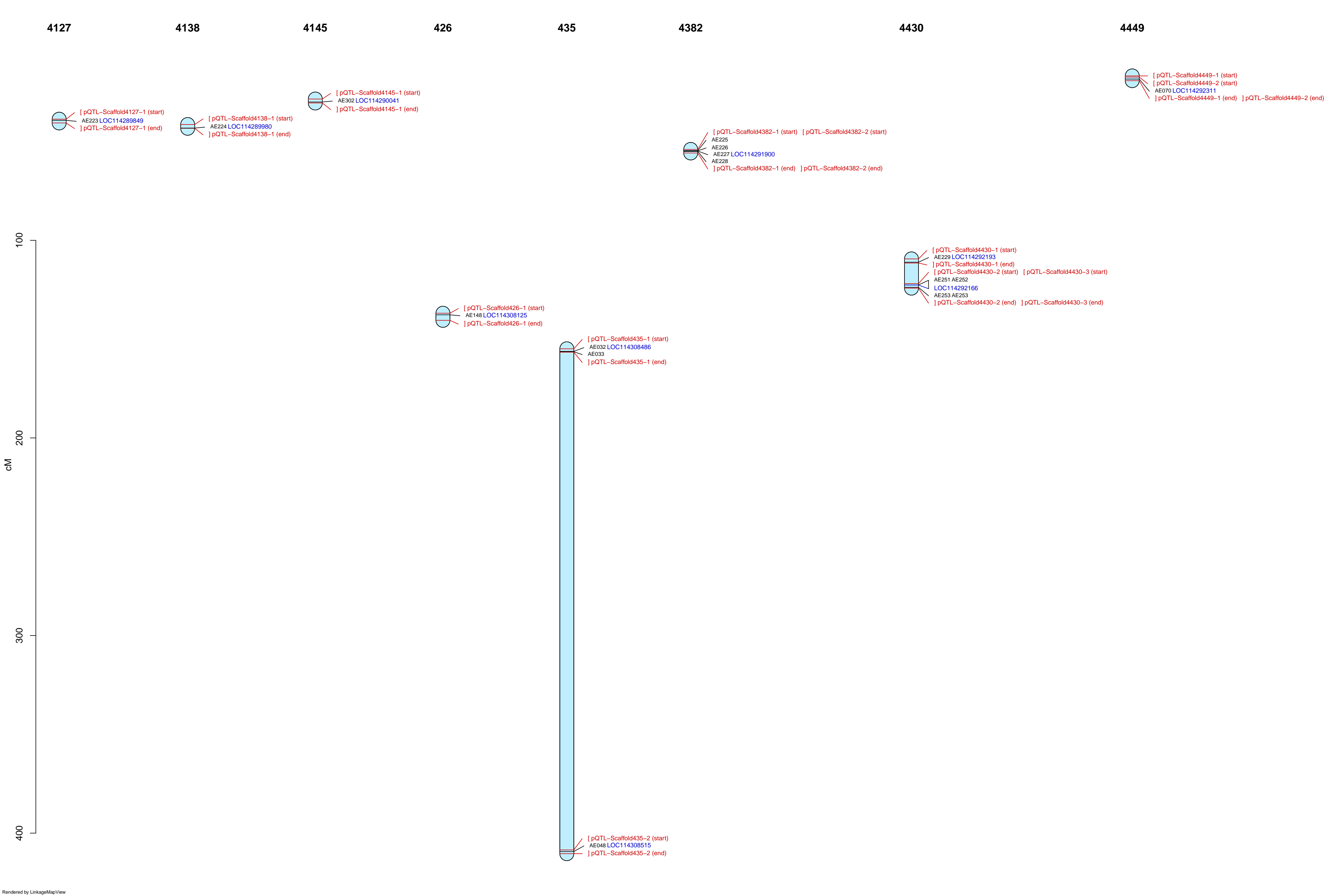

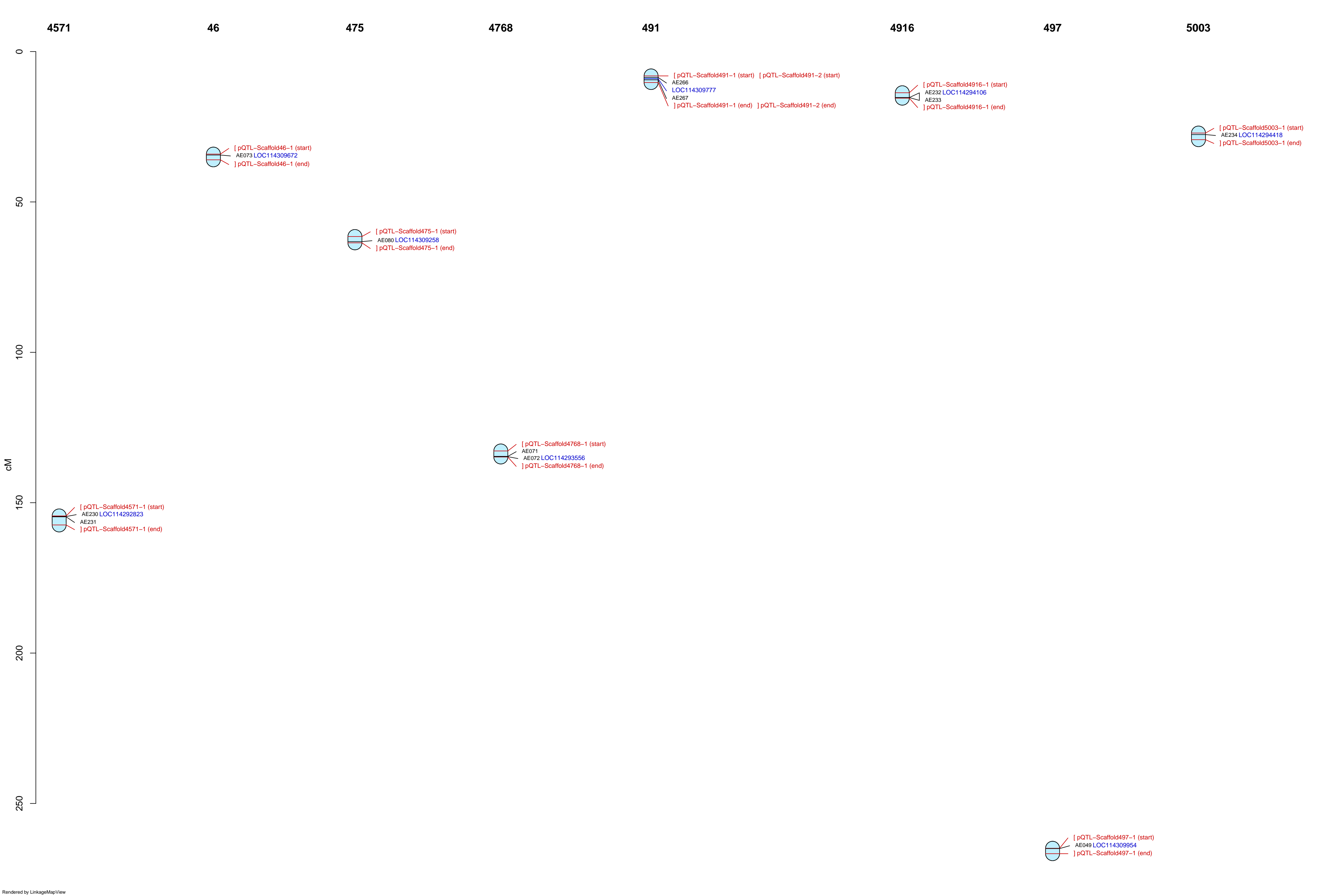

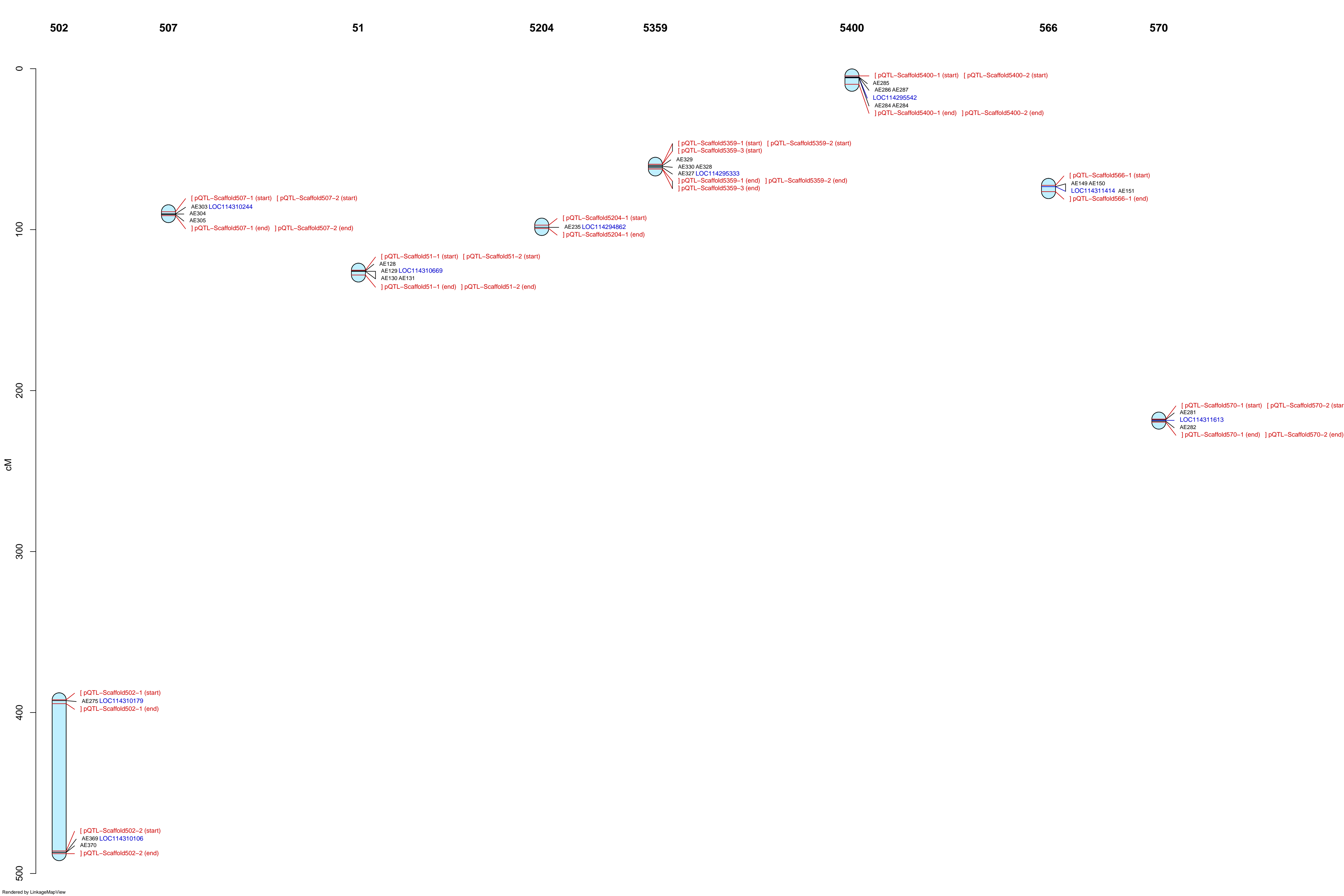

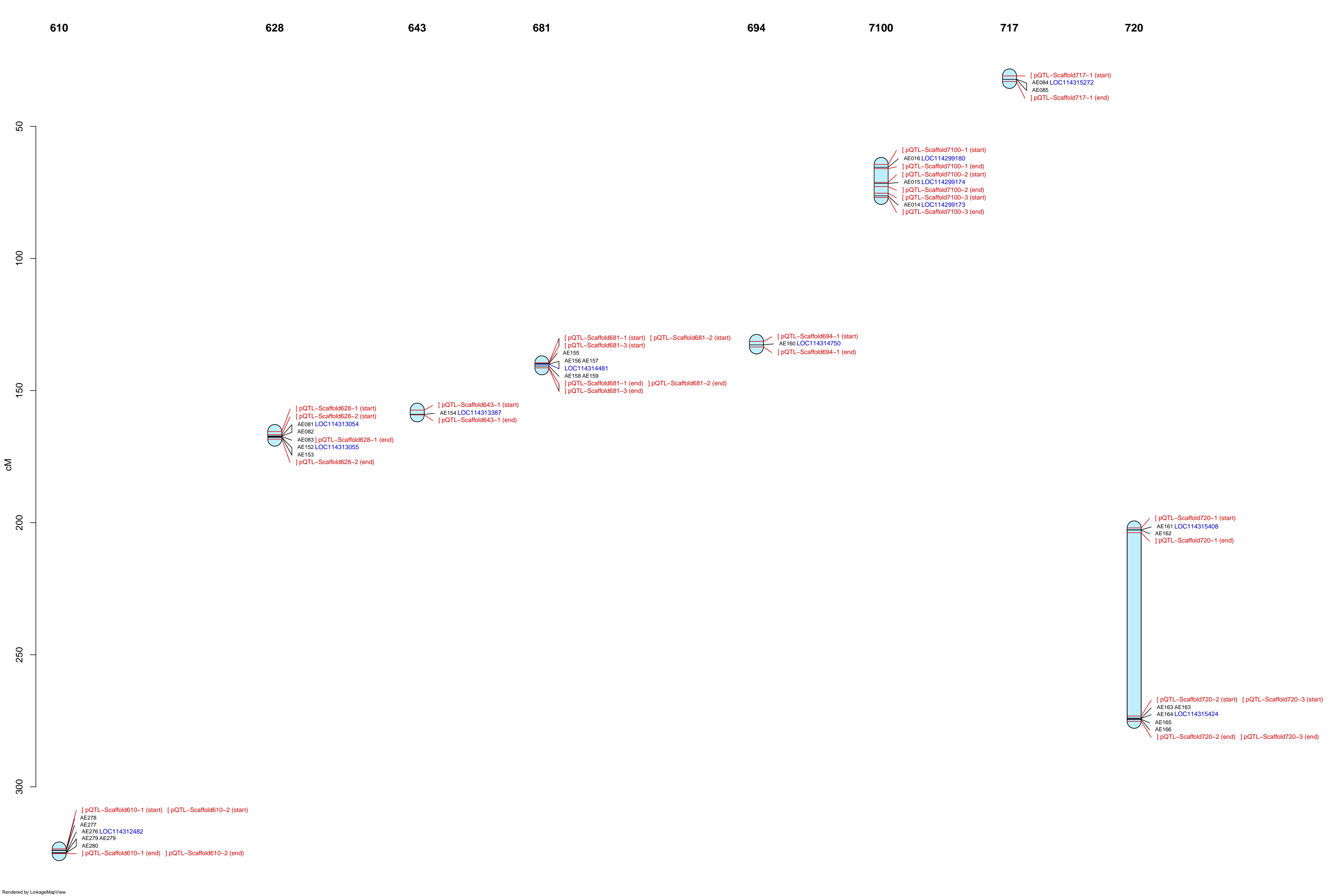

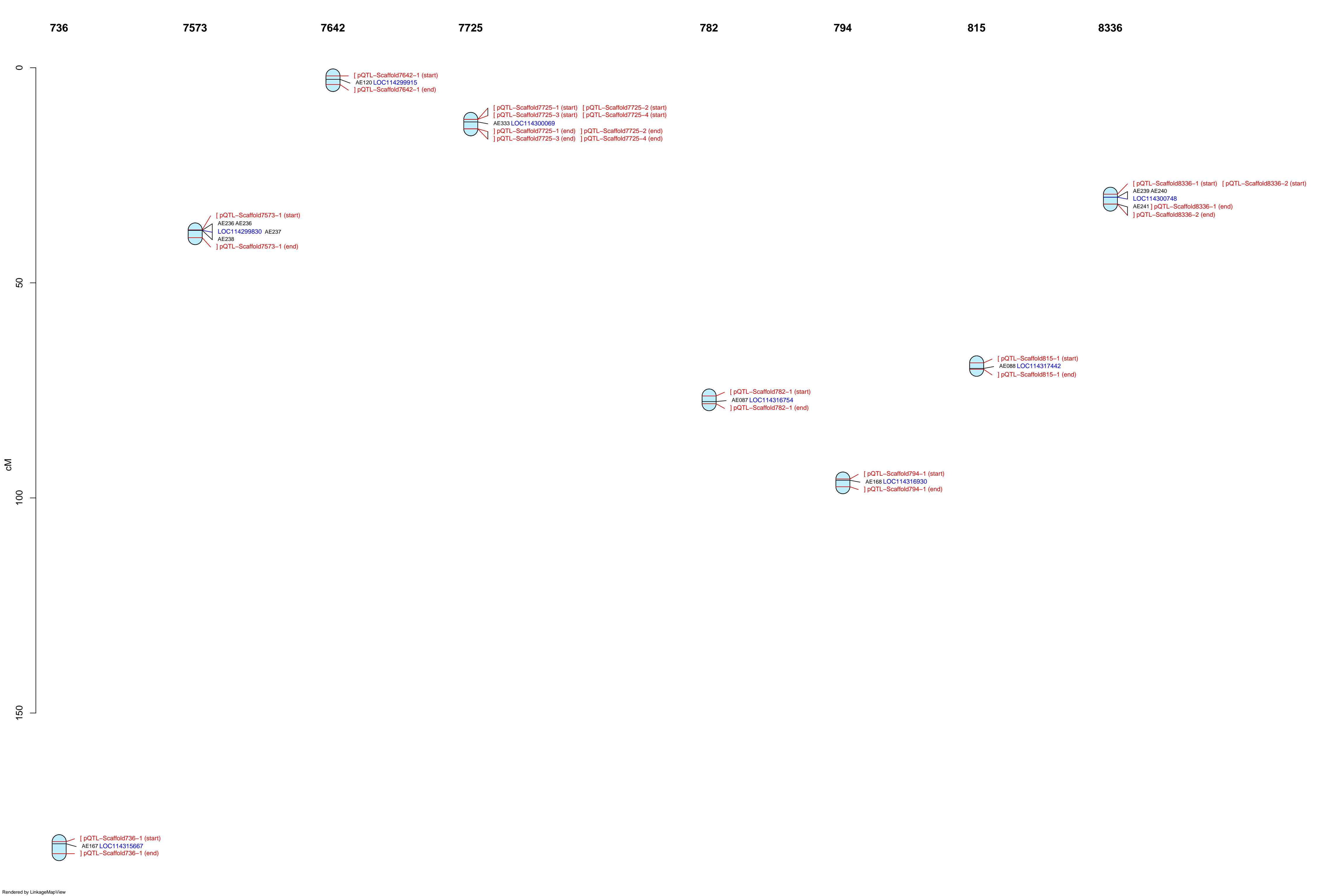

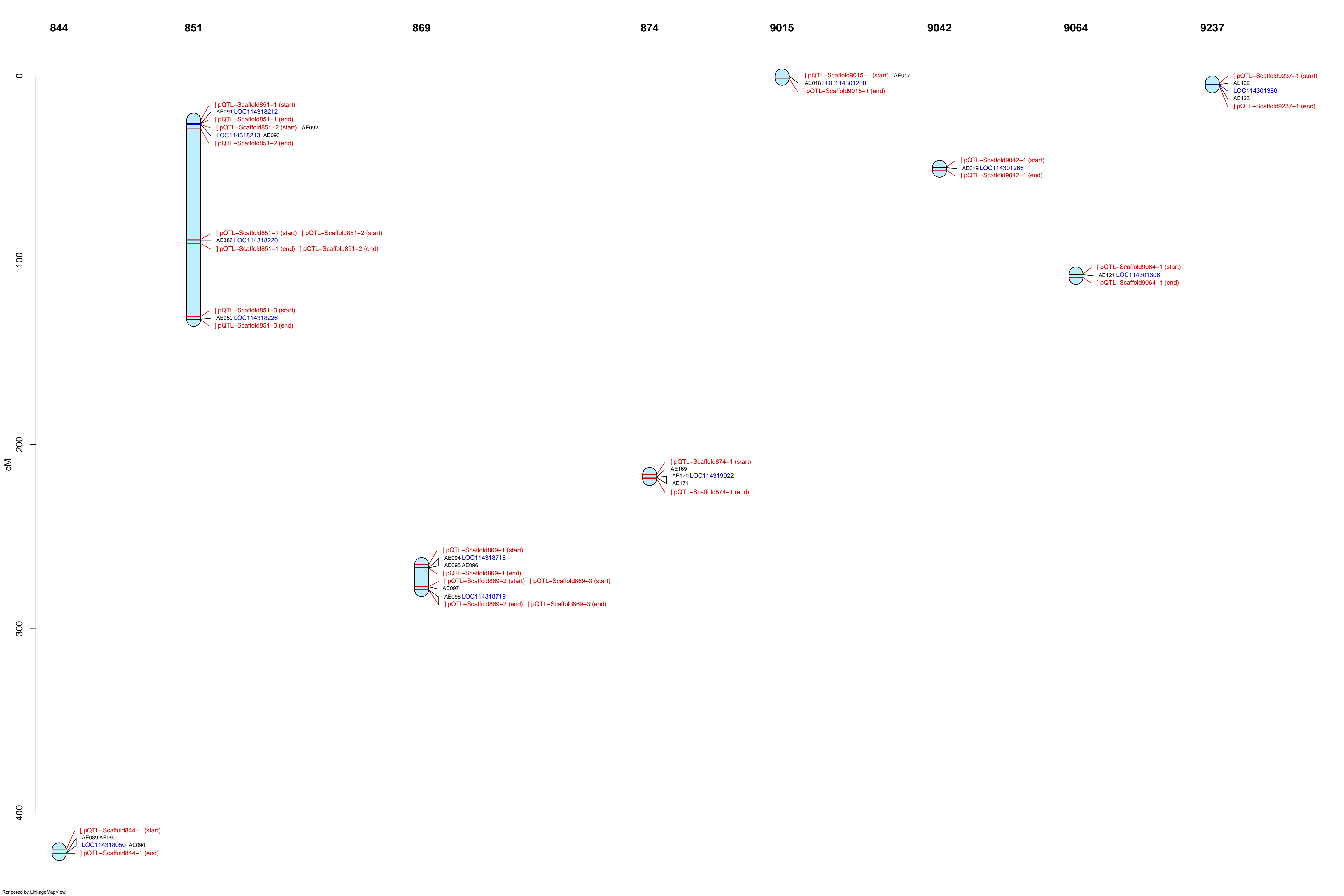

9306

974

979

981

985

996

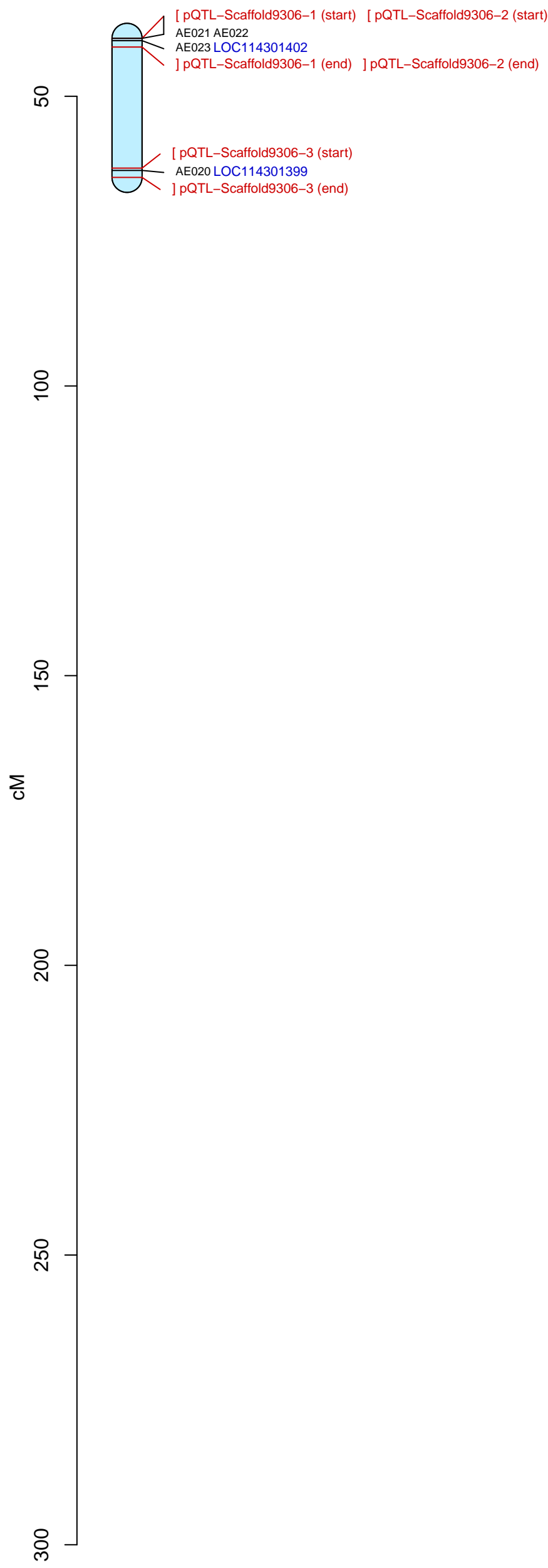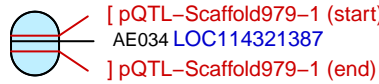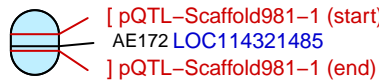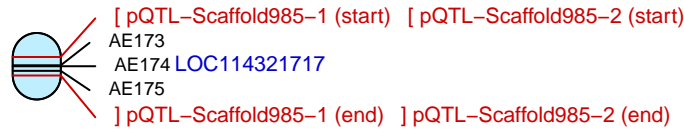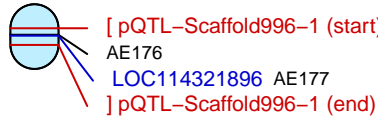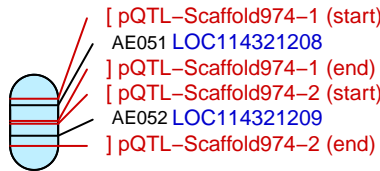

Supplement: Supplementary file 1 [file plants-15-00454-s001.zip › Supplementary File S7.pdf]
